# The MDM2 Inhibitor Navtemadlin Arrests Mouse Melanoma Growth *In Vivo* and Potentiates Radiotherapy

**DOI:** 10.1158/2767-9764.CRC-22-0053

**Published:** 2022-09-28

**Authors:** Katrine Ingelshed, Diana Spiegelberg, Pavitra Kannan, Linnéa Påvénius, Jessica Hacheney, Long Jiang, Silke Eisinger, Danai Lianoudaki, Dilraj Lama, Francisca Castillo, Cecilia Bosdotter, Warren W. Kretzschmar, Omayma Al-Radi, Nicolas Fritz, Eduardo J. Villablanca, Mikael C. I. Karlsson, Fredrik Wermeling, Marika Nestor, David P. Lane, Saikiran K. Sedimbi

**Affiliations:** 1 Department of Microbiology, Tumor and Cell Biology, Karolinska Institutet, Stockholm, Sweden.; 2 Department of Immunology, Genetics and Pathology, Uppsala University, Uppsala, Sweden.; 3 Department of Surgical Sciences, Uppsala University, Uppsala, Sweden.; 4 Division of Rheumatology, Department of Medicine Solna, Karolinska University Hospital and Karolinska Institutet, Stockholm, Sweden.; 5 Center for Molecular Medicine, Stockholm, Sweden.; 6 Division of Immunology and Allergy, Department of Medicine Solna, Karolinska University Hospital and Karolinska Institutet, Stockholm, Sweden.; 7 Vanadis Diagnostics, PerkinElmer Inc, Sollentuna, Sweden.

## Abstract

The tumor suppressor protein p53 is mutated in close to 50% of human tumors and is dysregulated in many others, for instance by silencing or loss of p14^ARF^. Under steady-state conditions, the two E3 ligases MDM2/MDM4 interact with and inhibit the transcriptional activity of p53. Inhibition of p53–MDM2/4 interaction to reactivate p53 in tumors with wild-type (WT) p53 has therefore been considered a therapeutic strategy. Moreover, studies indicate that p53 reactivation may synergize with radiation and increase tumor immunogenicity. *In vivo* studies of most MDM2 inhibitors have utilized immunodeficient xenograft mouse models, preventing detailed studies of action of these molecules on the immune response. The mouse melanoma cell line B16-F10 carries functional, WT p53 but does not express the MDM2 regulator p19^ARF^. In this study, we tested a p53-MDM2 protein–protein interaction inhibitor, the small molecule Navtemadlin, which is currently being tested in phase II clinical trials. Using mass spectrometry–based proteomics and imaging flow cytometry, we identified specific protein expression patterns following Navtemadlin treatment of B16-F10 melanoma cells compared with their p53 CRISPR-inactivated control cells. *In vitro*, Navtemadlin induced a significant, p53-dependent, growth arrest but little apoptosis in B16-F10 cells. When combined with radiotherapy, Navtemadlin showed synergistic effects and increased apoptosis. *In vivo*, Navtemadlin treatment significantly reduced the growth of B16-F10 melanoma cells implanted in C57Bl/6 mice. Our data highlight the utility of a syngeneic B16-F10 p53^+/+^ mouse melanoma model for assessing existing and novel p53-MDM2/MDM4 inhibitors and in identifying new combination therapies that can efficiently eliminate tumors *in vivo*.

**Significance:**

The MDM2 inhibitor Navtemadlin arrests mouse tumor growth and potentiates radiotherapy. Our results support a threshold model for apoptosis induction that requires a high, prolonged p53 signaling for cancer cells to become apoptotic.

## Introduction

The tumor suppressor protein p53 regulates multiple cellular processes such as the response to cellular stresses, cell division, apoptosis, DNA repair, and neoplastic transformation among others (1). Under steady-state conditions, p53 is regulated by the two E3 ligases MDM2 (HDM2 in human) and MDM4/X. While MDM2 can either monoubiquitinate p53, promoting nuclear export, or polyubiquitinate p53, inducing proteasome-mediated degradation, MDM4/X binds to the N-terminal transactivation domain of p53 and prevents its activity (2). Furthermore, MDM2 and MDM4/X can form dimers and oligomers with very high ligase activity. Approximately 50% of human cancers carry mutations in the p53 gene, primarily in the DNA-binding domain, which results in a nonfunctional p53 protein permitting the development of malignancy (3). In cancers with intact p53, a variety of mechanisms, including silencing or loss of p19^ARF^ (p14^ARF^ in human) or enhanced expression of MDM2 or MDM4 (4), serve to ablate the p53 response.

In tumors with wild-type (WT) p53, reactivating p53 by inhibiting p53-MDM2/4 protein–protein interaction (PPI) has therefore been considered a therapeutic strategy, and several small molecules and/or stapled peptides developed for this purpose have shown varying degrees of success in preclinical models and clinical trials (reviewed in ref. 5). Nutlin-3, a small molecule that binds MDM2 and induces a strong p53 response, has been extensively used to study p53-MDM2 PPI inhibition (6). Furthermore, Nutlin-3 has been used to identify the direct downstream targets of p53 (7). Despite its extensive use in p53 research, Nutlin-3 has been reported to have several effects in addition to inhibiting MDM2 (8). New p53-MDM2 PPI inhibitors have since been designed, for instance, RG7112 and Idasanutlin, which are in clinical trials (9, 10). Small molecule AM-8553 (11) was designed on the basis of the MDM2 binding mode of known MDM2 inhibitors. Further modifications of AM-8553 lead to the development of Navtemadlin (previously referred to as AMG 232 and KRT-232, Kartos Therapeutics), a very potent MDM2 inhibitor both *in vitro* and *in vivo* (12). In xenograft models, Navtemadlin induced p53 and its downstream targets p21, PUMA, and MDM2 in human SJSA osteosarcoma cells. Furthermore, Navtemadlin inhibited tumor growth in a dose-dependent manner and displayed synergistic effects with chemotherapeutic drugs Cisplatin, Carboplatin, and Doxorubicin (13). Navtemadlin has been reported to be the most potent MDM2 inhibitor to date and to induce robust tumor growth inhibition in WT p53 carrying cells, even when they harbored other oncogenic mutations. Apart from small molecules, stapled peptides that bind and inhibit both MDM2 and MDM4 have been developed over the past decade and show great promise in preclinical and clinical trials (14, 15).

Close to 80% of human patients with melanoma carry WT p53 (16, 17). However, overexpression of HDM2 (18) and loss of p14^ARF^ (19) are two mechanisms that negatively regulate p53 in melanoma. The mouse B16-F10 melanoma cells lack p19^ARF^ and p16^INK4A^ as a result of a deletion (20). p16^INK4A^ inactivates cyclin-dependent kinase (CDK) 4/6 and inhibits cell-cycle progression from G_1_- to S-phase while p19^ARF^ binds MDM2 inhibiting its E3 ligase activity, preventing p53 degradation, resulting in cell-cycle arrest in G_1_ and G_2_ phases (19). Reactivation of p53 in human and mouse melanoma cells either by genetic manipulation or by Nutlin-3 resulted in cell-cycle arrest *in vitro* and tumor growth reduction *in vivo*. In uveal melanoma cells, Nutlin-3 demonstrated growth inhibition when combined with topoisomerase inhibitor Topotecan (21). In B16-F10 cells, Nutlin-3a demonstrated a synergistic effect on growth inhibition *in vitro* and *in vivo* in combination with p19^ARF^ and interferon-β gene transfer approaches (22, 23).

Navtemadlin is very potent when compared with other p53-MDM2 PPI inhibitors such as RG7112, SAR299155, and Idasanutlin and has a dose-dependent antitumor activity *in vitro* in over 20 human cancer cell lines expressing WT p53 and several xenograft models (13). Furthermore, Navtemadlin showed a synergistic effect when combined with radiation (24). Navtemadlin, even though tested extensively in human cancer cell lines and xenograft models, has been reported to be ineffective (40-fold less biochemical potency) on mouse tumor cells (13). This resulted in a lack of further studies assessing the role of Navtemadlin in syngeneic tumor models with an intact immune system. To test the hypothesis if Navtemadlin is indeed ineffective on mouse tumor cells, we chose the B16-F10 mouse melanoma model as it is known to lack p19^ARF^ and carry WT p53. Unexpectedly, our data demonstrate that Navtemadlin potently induces cell-cycle arrest in mouse tumor cells in a p53-dependent manner. The combination with radiotherapy increased p53 protein concentration and induced high levels of apoptosis rather than cell-cycle arrest. Proteomics and imaging flow cytometry analyses indicate that Navtemadlin is highly specific in mouse models, as p53^−/−^ cells treated with Navtemadlin did not display any changes in protein expression. *In vivo*, Navtemadlin induced tumor growth reduction in immune-competent C57Bl/6 mice.

## Materials and Methods

### Cell Lines and Cell Culture

B16-F10 murine melanoma cells (ATCC-CRL-6475) were purchased from ATCC. B16-F10 p53^−/−^ cells were generated by inactivating *Trp53* using CRISPR-Cas9. In short, the *Trp53* targeting gRNA oligo (ACAAAATTACAGACCTCGGG) was cloned into pX459 plasmid (https://www.addgene.org/62988/). The plasmids were then transfected into B16-F10 cells using Lyovec (Invivogen) using the manufacturer's protocol. Transfected cells were selected for resistance to puromycin (5 μg/mL) for 24 hours. Surviving cells were seeded at one cell per well to generate single-cell clones. Following expansion, different clones of cells were collected for genomic DNA extraction and PCR amplification of the targeting sequence (Forward: TGGTGATGGTAAGCCCTCAAC, Reverse: TGGTATACTCAGAGCCGGCC). PCR amplicons were sequenced using Sanger sequencing and the sequencing data were analyzed by ICE software online (https://ice.synthego.com). B16-F10 p53^−/−^ clone 8, with complete *Trp53* inactivation, was used for all experiments (Supplementary Fig. S1).

Following p53 deletion, both p53^+/+^ and p53^−/−^ (clone 8) were maintained in RPMI-1640 (R8758, Sigma-Aldrich) with 10% heat-inactivated FBS (SV30160.03, Hyclone) and 100 U/mL penicillin and 100 μg/mL streptomycin (5140-122, Gibco). YUMM 1.7 murine melanoma cells (ATCC-CRL-3362) were obtained from ATCC and maintained in DMEM/F12 (31331-028, Gibco) supplemented with 10% heat-inactivated FBS (SV30160.03, Hyclone), 100 U/mL penicillin, and 100 μg/mL streptomycin (5140-122, Gibco), 1 × MEM Non-Essential Amino Acids (11140-050, Gibco) and 15 mmol/L HEPES (15630-056, Gibco). CT26.WT murine colon carcinoma cells (ATCC-CRL-2638) were purchased from ATCC and cultured in RPMI-1640 (R8758, Sigma-Aldrich) supplemented with 10% heat-inactivated FBS (SV30160.03, Hyclone) and 100 U/mL penicillin and 100 μg/mL streptomycin (5140-122, Gibco). NIH/3T3 murine embryonic fibroblast cells (CRL-1658), purchased from ATCC were cultured in DMEM (D6429, Sigma-Aldrich) containing 10% heat-inactivated bovine calf serum (12133C, Sigma-Aldrich) and 100 U/mL penicillin and 100 μg/mL streptomycin (5140-122, Gibco). All cell lines used in this study were purchased from ATCC that performs authentication. At receipt, all cell lines were expanded and frozen at low passage numbers. Prior to use in experiments, cells were thawed and allowed to adjust to culture conditions for at least 1 week. The cells were subcultured at least twice per week and maintained in culture for less than five months. *Mycoplasma* testing of all the cell lines used in this study was performed once every 2 months using the MycoAlert Plus Detection kit (LT07-710, Lonza) according to the manufacturer's instructions.

### *In Vitro* Treatments

Optimal seeding density was determined by following the growth of the cells seeded at varying densities (in a 96-well flat bottom plate) in the IncuCyte S3 live imaging system. A seeding density of 1 × 10^3^ cells/well was chosen for B16-F10 p53^+/+^, B16-F10 p53^−/−^, YUMM 1.7, and CT26.WT cells and 1.5 × 10^3^ for NIH/3T3 cells/well was chosen for all live cell imaging experiments. AMG 232 (2639, Axon Medchem), referred to as Navtemadlin, was dissolved in DMSO (D2650, Sigma-Aldrich) to obtain 40 mmol/L stock solutions. Further dilutions were made in cell culture medium, based on the experimental setup. DMSO was used as the vehicle control. For irradiation experiments, p53^+/+^ and p53^−/−^cells were seeded 24 hours prior to irradiation with 2, 4, or 6 Gy using a X-RAD iR-225 Biological X-ray Irradiator at a dose rate of 1 Gy/minute (Precision X-Ray) and Navtemadlin was added 2 hours after irradiation.

### Mice

Eight to nine weeks old, female C57Bl/6JBom mice were purchased from Taconic and maintained under specific pathogen-free conditions at the Karolinska Institutet (Stockholm, Sweden). A total of 1 × 10^5^ B16-F10 cells in 100 μL PBS (D8537, Sigma-Aldrich) were mixed with 150 μL cold Matrigel (356231, Corning) and p53^+/+^ cells were injected subcutaneously on the right flank and p53^−/−^ cells were injected subcutaneously on the left flank. Navtemadlin was dissolved in PBS with a final concentration of 11% DMSO. A total of 20 mg/kg Navtemadlin or 11% DMSO was injected in 200 μL PBS intraperitoneally every 24 hours, starting 3 days after tumor implantation, when tumor pigmentation was visible through the skin of the mice. Tumor volume was measured at regular intervals using digital calipers. Tumor volume was calculated with the equation ((small side) squared) × (long side) × 0.5236). Mice were weighed daily and stools were observed on a daily basis. At endpoint, day 12 or 13 (maximum tumor size 1,000 mm^3^), mice were euthanized using CO_2_. All experiments were reviewed and approved by the responsible Institutional Ethical Committee (the North Stockholm District Court).

### Bone Marrow Analysis

At endpoint, after 10 days treatment, femur and tibia were collected and placed on ice in PBS. The bones were cut and flushed with PBS. Red blood cells were lysed with an ammonium chloride–based RBC lysis buffer (MIK 3242, Clinical microbiology Karolinska University hospital). The cells were counted with an automated cell counter and 10 × 10^6^ cells from each sample were stained. Dead cells were stained with Live/Dead Fixable Aqua Dead Cell Stain Kit (L34966 Invitrogen) at a 1:400 dilution in PBS for 10 minutes in the dark at room temperature. After washing with PBS supplemented with 2 mmol/L Ethylenediaminetetraacetic acid (EDTA) and 1% FBS (FACS buffer), the cells were stained with anti CD34-Alexa 700, 1:50 (RAM34, BD) in FACS buffer for 2 hours at 4°C. The samples were washed with FACS buffer and stained with a cocktail of Lin-Biotin, 1:200 (catalog no. 130-092-613, Miltenyi Biotec), Sca-1-PECy-7, 1:200 (D7, BioLegend), c-kit-APC, 1:200 (2B8, BD), and CD16/34-BV605, 1:200 (2.4 G2, BD) in FACS buffer for 30 minutes on ice. The samples were washed with FACS buffer and additionally stained with Streptavidin-APC Cy7, 1:1,000 (catalog no. 405208, BioLegend) for 30 minutes on ice. After a final wash with FACS buffer, the samples were acquired on a BD LSR II Flow cytometer. The data were analyzed with FlowJo v10.8 (Treestar).

### Tumor-infiltrating Immune Cell Analysis

Tumors were harvested 2 hours after the last treatment and placed on ice in DMEM (D6429, Sigma-Aldrich) supplemented with 2% heat-inactivated FBS, 1 × MEM Non-Essential Amino Acids (11140-050, Gibco), 1 × MEM amino acids (11130-036, Gibco), 15 mmol/L HEPES (15630-056, Gibco), 100 μg/mL DNAse I (11284932001, Roche), and 150 μg/mL Liberase (05401127001, Roche). The tumors were mechanically dissociated with scissors and then incubated for 30 minutes at 37°C to activate the collagenases and another 10 minutes on ice to continue the tissue dissociation. Each sample was further mechanically dissociated through 70 micron strainers and washed with PBS supplemented with 0.5% BSA (A9647, Sigma-Aldrich), 2 mmol/L EDTA (E177, Amresco), 2 mmol/L l-glutamine (25030081, Gibco), 1 mmol/L Sodium Pyruvate (11360-070, Gibco), 1 × MEM Non-Essential Amino Acids (11140-050, Gibco), 1 × MEM Amino acids (11130-036, Gibco), and 4.5 g/L dextrose (G8270, Sigma-Aldrich). Erythrocytes were lysed at room temperature for 4 minutes with an ammonium chloride–based RBC lysis buffer (MIK 3242, Clinical microbiology Karolinska University hospital). Washed single-cell suspensions in PBS were incubated for 10 mins at room temperature with Fc block, 1:400 (2.4G2, BD Biosciences) and Live/Dead Fixable Aqua Dead Cell Stain Kit, 1:400 (L34966 Invitrogen). The samples were washed with PBS supplemented with 1% heat-inactivated FBS (SV30160.03, Hyclone) and 2 mmol/L EDTA (E177, Amresco), (FACS buffer) and extracellularly stained in FACS buffer, in two panels, for 30 minutes on ice. Tumor-infiltrating myeloid cells panel: CD45-BV786, 1:300 (30-F11, BD Biosciences), CD11b-BV711, 1:400 (M1/70, BD Biosciences), F4/80-PerCPCy5.5, 1:100 (BM8 BioLegend), I-A/I-E-BV421, 1:200 (M5/114.15.2, BD Biosciences), CD11c-PECy7, 1:200 (HL3, BD Biosciences), Ly-6C-APC/Fire750, 1:200 (HK1.4, BioLegend), Ly-6G-BV605, 1:200 (1A8, BioLegend), TCRβ-BV510, 1:200 (H57-597, BD Biosciences), B220-BV510, 1:100 (RA3-6B2, BD Biosciences) NK.1.1-BV510, 1:200 (PK136, BD Biosciences). Tumor-infiltrating lymphocyte panel: CD45-BV786, 1:300 (30-F11, BD Biosciences), CD3ε-PerCPCy5.5, 1:50 (145-2C11, BioLegend), B220-PE, 1:100 (RA3-6B2, BioLegend), NK1.1-Alexa flour 488, 1:200 (PK136, BioLegend), CD8-APC, 1:200 (53-6.7, BioLegend), CD4-BV605, 1:200 (RM4-5, BioLegend), CD44-BV421, 1:200 (IM7 BioLegend), CD25-PECy7, 1:200 (PC61, BD Biosciences), CD62L-BV711, 1:200 (Mel-14, BioLegend), CD11b-BV510, 1: 400 (M1/70, BD Biosciences). After extracellular staining, all samples were fixed for 30 minutes and stained intracellularly for 30 minutes using the FoxP3 staining kit (Thermo Fisher Scientific) according to manufacturer's instructions. Tumor-infiltrating myeloid cells panel: Arginase I-Alexa Fluor 488, 1:100 (A1exF5, Invitrogen). Tumor-infiltrating lymphocyte panel: Foxp3-Biotin 1:100 (FJK-16s, Invitrogen) and Streptavidin-APC/Fire750, 1:100 (catalog no. 405250, BioLegend). The samples were washed and resuspended in FACS buffer. Samples were recorded in a BD LSR II Flow cytometer. The data were analyzed with FlowJo v10.8. (Treestar).

### Fecal Lipocalin-2 ELISA

Feces were collected directly from the colon on the last experimental day and fecal weight was determined immediately after collection. Feces samples were dissolved in PBS containing 0.1% Tween 20 to a final concentration of 100 mg/mL. For an optimal homogenization, samples were disrupted with a pellet pestle and further vortexed at 1,400 × *g* for 5 minutes. Next, samples were centrifuged at 12,000 rpm for 10 minutes at 4°C. Supernatant was collected into a new tube and stored at −20°C until analysis. DuoSet Mouse Lipocalin-2/NGAL ELISA (R&D Systems) was used to determine the concentration of Lipocalin-2 (LCN-2) in the fecal supernatants (diluted in 1:50 in 1% BSA in PBS) following manufacturer's instructions. The optical density was read at 450 nm using a SpectraMax iD3 Multi-mode microplate reader (Molecular Devices). Final concentrations of LCN-2 were determined using a standard curve.

### Live Cell Imaging

To investigate the effect of Navtemadlin on cells in exponential growth, B16-F10 p53^+/+^, p53^−/−^ cells, YUMM 1.7 cells and CT26.WT cells (1,000 cell/well) and NIH/3T3 fibroblast cells (1,500 cells/well) were seeded in 96-well plates (TPP) in 200 μL culture medium/well. The cells were allowed to adhere overnight and Navtemadlin or DMSO control, in fresh RPMI medium was added to each well (200 μL/well). All treatments were performed in a minimum of triplicates. The cells were imaged at 37°C and 5% CO_2_ every second hour in the IncuCyte S3 live cell imaging system. 10 × phase images were collected and the confluence was analyzed using the IncuCyte software.

### Cell-cycle Analysis

The cell-cycle phases were evaluated using the Click-iT Plus EdU Alexa Fluor 488 Flow cytometry assay kit (C10633, Thermo Fisher Scientific) according to the manufacturer's protocol. In short, B16-F10 p53^+/+^ and p53^−/−^ cells were treated with Navtemadlin in T25 flasks (TPP) for 24, 48, and 72 hours. A total of 10 μmol/L (final concentration) of EdU was added 1 hour before harvesting the cells with enzyme-free, PBS-based cell dissociation buffer (13151-014, Gibco, Life Technology). All staining steps were performed in V-bottomed 96-well plates. Cells were fixed in 100 μL Fix/Perm buffer (eBioscience Foxp3 staining buffer set 00-5523-00, Thermo Fisher Scientific) at 4°C for 30 minutes. After washing with 1% BSA-PBS cells were incubated with Click IT plus reaction cocktail for 30 minutes at room temperature. Following a final wash with 1 × Saponin perm buffer, cells were resuspended in 1 × Saponin perm buffer containing FxCycle Violet (1:1,000 dilution, Thermo Fisher Scientific). A minimum of 10,000 single cells were acquired at low speed on a BD LSR II Flow cytometer. The data were analyzed with FlowJo v10.6.1 (Treestar).

### Proteomics

B16-F10 p53^+/+^ and p53^−/−^ cells were seeded as triplicates in T75 flasks (Falcon) at densities corresponding with exponential growth during the treatment time. After allowing the cells to attach overnight, cells were treated with fresh culture medium containing 1.5 μmol/L of Navtemadlin, or DMSO. After 6, 48, and 72 hours treatment, cells were washed with PBS and detached with Trypsin/EDTA. The cells were washed with PBS and the pellet was lysed at −80°C. Cell pellets were dissolved in 300 μL Lysis buffer (4% SDS, 25 mmol/L HEPES pH 7.6, 1 mmol/L DTT), heated to 95°C and sonicated. The total protein amount was estimated (Bio-Rad DC). Samples were then prepared for mass spectrometry analysis using a modified version of the Filter Aided Sample Preparation (FASP) protocol digestion (25), where proteins were digested by trypsin (Thermo Fisher Scientific). In brief, 200 μg of protein per sample was mixed with 1 mmol/L DTT, 8 mol/L urea, 25 mmol/L HEPES pH 7.6 in a centrifugation filtering unit with a 10 kDa cutoff (Nanosep Centrifugal Devices with Omega Membrane, 10 k). The samples were then centrifuged for 15 minutes at 14,000 × *g*, followed by another addition of the 8 mol/L urea buffer and centrifugation. Proteins were alkylated by 25 mmol/L IAA, in 8 mol/L urea, 25 mmol/L HEPES pH 7.6 for 10 minutes, centrifuged, followed by two more additions and centrifugations with 8 mol/L urea, 25 mmol/L HEPES pH 7.6. Protein samples were digested on the filter, using trypsin (Thermo Fisher Scientific), enzyme: protein ratio 1:50 in 50 mmol/L HEPES was added and incubated overnight at 37°C. After digestion, the filter units were centrifuged for 15 minutes, 14,000 × *g*, followed by another centrifugation with 50 μL MilliQ water. Peptides were collected and the peptide concentration determined (Bio-Rad DC Assay). A total of 1 μL of each sample was taken out for digestion check by LC/MS-MS analysis to determine the percentage missed cleavages. Before labeling, samples were pH adjusted using TEAB pH 8.5 (100 mmol/L final conc.), 80 μg of peptides were labeled with isobaric TMT tags (TMT10plex reagent) according to the manufacturer's protocol (Thermo Fisher Scientific). Labeling efficiency was determined by LC/MS-MS. After the samples passed labeling efficiency test, they were pooled for each TMT 10plex.

For the sample clean-up step, a solid phase extraction (SPE strata-X-C, Phenomenex) was performed and purified samples were dried in a SpeedVac.

The labeled samples were separated by immobilized pH gradient–isoelectric focusing (IPG-IEF) on 3–10 strips as described previously (26). Briefly, 400 μg of the dried peptide sample pools were subjected to peptide IEF-IPG (isoelectric focusing by immobilized pH gradient) in the pI range 3–10. Peptide samples were dissolved in 250 μL rehydration solution containing 8 mol/L urea and 1% IPG pharmalyte pH 3–10 (GE Healthcare) and allowed to adsorb to the gel strip by swelling overnight. The peptides were focused on the gel strip and the peptides were passively eluted into 72 contiguous fractions with MilliQ water/35% ACN/35% ACN + 0.1% FA using an in-house constructed IPG extractor robotics (GE Healthcare Bio-Sciences AB, prototype instrument) into a 96-well plate (V-bottom, Greiner product #651201), which was then dried in a SpeedVac. The resulting fractions were dried and kept at −20°C.

Online LC/MS was performed using a Dionex UltiMate 3000 RSLCnano System coupled to a Fusion mass spectrometer (Thermo Fisher Scientific). Each of the 72-well plates was dissolved in 20 μL solvent A and 10 μL were injected. Samples were trapped on a C18 guard-desalting column (Acclaim PepMap 100, 75 μm × 2 cm, nanoViper, C18, 5 μm, 100Å), and separated on a 50-cm-long C18 column (Easy spray PepMap RSLC, C18, 2 μmol/L, 100Å, 75 μmol/L × 50 cm). The nanocapillary solvent A was 99.9% water and 0.1% formic acid; and solvent B was 5% water, 95% acetonitrile, and 0.1% formic acid. At a constant flow of 0.25 μL minute^−1^, the curved gradient went from 6% to 8% B up to 40% B in each fraction in a dynamic range of gradient length followed by a steep increase to 100% B in 5 minutes. FTMS master scans with 60,000 resolution (and mass range 300–1,500 m/z) were followed by data-dependent MS-MS (30,000 resolution) on the top five ions using higher energy collision dissociation (HCD) and collision-induced dissociation (CID) at 35% normalized collision energy. Precursors were isolated with a 2 m/z window. Automatic gain control targets were 1 × 10^6^ for MS1 and 1 × 10^5^ for MS2. Maximum injection times were 100 ms for MS1 and 100 ms for MS2. The entire duty cycle lasted approximately 2.5 seconds. Dynamic exclusion was used with 30 seconds duration. Precursors with unassigned charge state or charge state 1 were excluded. An underfill ratio of 1% was used.

### Peptide and Protein Identification

The MS raw files were searched using SequestHT-Target Decoy PSM Validator under the software platform Proteome Discoverer 1.4 (Thermo Fisher Scientific) against mouse Swissprot database (released March 2019) and filtered to a 1% FDR cutoff. We used a precursor ion mass tolerance of 10 ppm, and product ion mass tolerances of 0.02 Da for HCD-FTMS and 0.8 Da for CID-ITMS. The algorithm considered tryptic peptides with maximum two missed cleavages; carbamidomethylation (C) and TMT-6plex as fixed modifications; oxidation (M), as variable modifications. Pathway analysis was performed with all the upregulated and downregulated genes using g:Profiler (27).

### Imaging Flow Cytometry

B16-F10 cells treated with Navtemadlin or DMSO control, were harvested and single-cell suspensions were incubated with Fc Block (clone 2.4G2, BD Biosciences) for 10 minutes at room temperature to reduce unspecific antibody binding. Intracellular staining to detect p53, p21, Bax, PUMA, BCL-2, and MCL-1 Ps159 was performed using the FoxP3 staining kit (Thermo Fisher Scientific) according to the manufacturer's instructions. Briefly, cells were fixed in fixation/permeabilization buffer for 30 minutes at 4°C and washed with permeabilization buffer. Fixed cells were then incubated for 30 minutes at 4°C with antibodies against intracellular proteins: p53-Alexa 647, 1:75 (1C12, Cell Signaling Technology); p21-Alexa 488, 1:100 (F-5);Bax-Alexa 595, 1:100 (B-9); PUMA-PE, 1:100 (B-6) from Santa Cruz Biotechnology; BCL-2-PE-Vio770, 1:10 (REA356, Miltenyi Biotec) and MCL-1 Ps159-PE, 1:50 (REA924, Miltenyi Biotec).

After washing, single-cell suspensions were resuspended in flow buffer (0.5% BSA, 2 μmol/L EDTA in PBS) containing the DNA stain FxCycle Violet (1:2,000 dilution, Thermo Fisher Scientific). A minimum of 30,000 cells were acquired on an ImageStreamX Mk II Imaging Flow Cytometer (Amnis corporation) equipped with 405, 488, 561, 642, and 785 nm lasers at 60 × magnification. Data analyses were performed using IDEAS Software (Amnis Corporation).

### Western Blotting

Whole lysates from B16-F10 p53^+/+^ and p53^−/−^ cells were probed for p53 and p21 expression. Cells were treated for 24 hours with 1.5 μmol/L Navtemadlin (to activate p53) or with DMSO control, lysed using 1 × Laemmli lysis buffer, heated at 95°C for 5 minutes, and sonicated (Qsonica Sonicators) for 30 seconds at 20% amplitude. After protein concentrations were determined using the DC Protein Assay (Bio-Rad), lysates were loaded (18 μg/lane) in a 4%–15% polyacrylamide gel (Mini-Protein TGX Stain-Free 12 well, Bio-Rad), and electrophoresed for 45 minutes at 150 V. Proteins were then transferred using a semidry method onto polyvinylidene difluoride membranes for 30 minutes according to manufacturer's instructions (Bio-Rad). Once membranes were blocked for 1 hour with blocking buffer (5% milk made in PBS containing 0.1% Tween-20), they were incubated overnight at 4°C with rabbit anti-mouse p53 (Abcam, EPR20416-124, 1:1,000) or rabbit anti-mouse p21 (Abcam, EPR18021, 1:1,000) primary antibodies made in blocking buffer. Membranes were subsequently washed in PBS containing 0.1% Tween-20, incubated for 1 hour at room temperature with goat anti-rabbit HRP secondary antibody (Dako, 1:1,000), and washed again. Protein expression was detected using chemiluminescence (Clarity Western ECL Substrate, Bio-Rad).

### Synergy Calculations

The growth rates of the B16-F10 WT cell cultures at each combination of Navtemadlin dose and radiation dose were calculated. The growth rate calculation was performed by extracting the growth curve in the linear response range of the method (after lag phase and before confluence). This was determined to be >24 hours and <72 hours for all combinations. Then, a linear regression was performed for each combination of doses in the linear interval, and the growth rate measured as the slope of the regression curve. The growth rates were normalized to the fastest growing combination. All data preparation was performed in R (v4.0.5), using RStudio (v1.3.1093). The SynergyFinder webpage (https://synergyfinder.org, visited May 2022) was used for synergy calculations on the growth rate data, and yielded dose–response curves, IC_50_ for each individual treatment, as well as ZIP/Loewe/Bliss synergy scores.

### Statistical Analysis

Unless otherwise indicated, a one-way or two-way ANOVA with Tukey test to correct for multiple comparisons was used to assess the statistical differences between experimental groups. A *P* value of <0.05 was considered statistically significant. Data analyses were performed using GraphPad PRISM software version 8.0. Because of heteroscedasticity and interdependency of variables, flow cytometry data measuring cell-cycle phases were analyzed using a negative binomial regression model as described previously (28), with model specification as counts ∼ phase * cell_type * treatment + experiment (R project). Imaging flow cytometry data measuring protein expression after treatment over time were analyzed using a linear mixed effects model; the best-fit model included time, concentration, and the interaction of time and concentration as fixed effects, and experiment as a random intercept with unequal variances (R project).

### Data Availability

The data generated in this study are available within the article and its Supplementary Data.

## Results

### Navtemadlin Induces Cell-cycle Arrest in B16-F10 Melanoma Cells in a p53-dependent Manner

**FIGURE 1:**
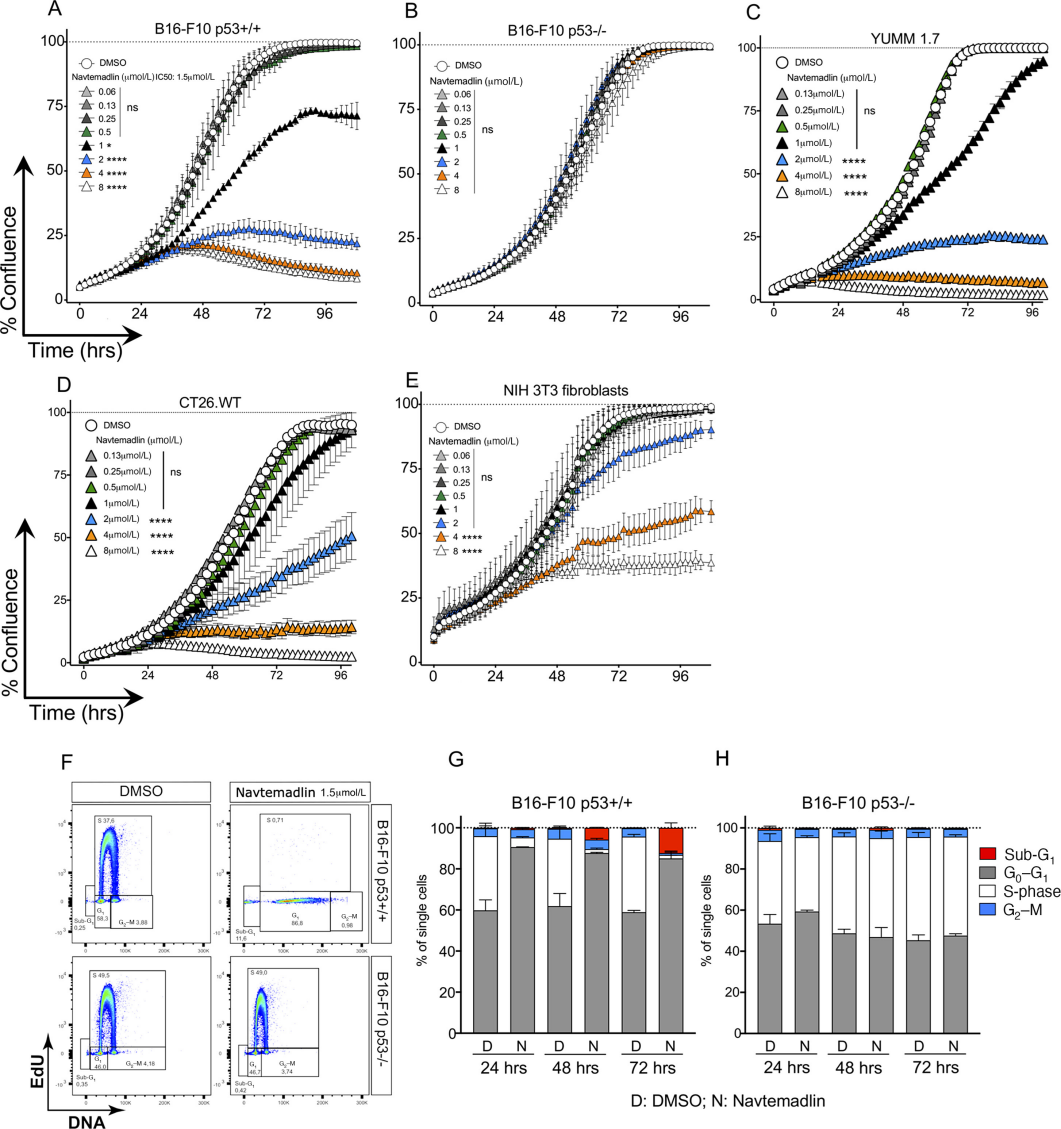
Navtemadlin inhibits murine cell growth in a p53-dependent manner. **A–E,** Cell growth, measured as % confluence over time, of B16-F10 p53^+/+^ and p53^−/−^ mouse melanoma cells, YUMM 1.7 mouse melanoma cells, CT26.WT mouse colon carcinoma cells, and NIH/3T3 mouse fibroblast cells treated with indicated concentrations of Navtemadlin or vehicle control was monitored for 96 hours (IncuCyte S3 system). **F,** Representative plots showing flow cytometry analyses of cell-cycle measuring incorporation of EdU into newly synthesized DNA after 72 hours treatment with 1.5 μmol/L Navtemadlin. DNA was stained with FxCycle. The gates were based on FMO controls. **G** and **H,** Cell-cycle phases of B16-F10 p53^+/+^ and p53^−/−^ cells 24, 48, and 72 hours after treatment with 1.5 μmol/L Navtemadlin based on EdU incorporation in proliferating cells. The data are representative of at least two experiments performed with triplicates. Mean ± SD. *, *P* <0.05; ****, *P* < 0.0001. One-way ANOVA.

Navtemadlin has been demonstrated to be highly potent in inducing p53 and cell-cycle arrest in human tumor cells carrying WT p53, but not in those that are p53 mutated or null (13). As it has previously been reported that Navtemadlin does not affect mouse tumor cells, we set out to test whether this indeed is the case by generating p53^−/−^ B16-F10 melanoma cells from the parent cell line p53^+/+^ B16-F10 using the CRISPR-Cas9 method (Supplementary Fig. S1A and S1B). Western blot analysis confirmed that p53 and its downstream target p21 were not expressed in p53^−/−^ B16-F10 cells following Navtemadlin treatment (Supplementary Fig. S1C). To study the effect of Navtemadlin on p53^+/+^ and p53^−/−^ B16F10 cells, we treated them with various concentrations of Navtemadlin for 96 hours where cell growth and confluence were tracked in real time using the live cell imaging system IncuCyte S3. Navtemadlin inhibited the growth of B16-F10 p53^+/+^ cells in a dose-dependent manner while the drug treatment did not affect B16-F10 p53^−/−^ cells even at the highest concentration tested. Significant growth arrest was observed with treatment doses of 1 μmol/L and above with an IC_50_ of 1.5 μmol/L (Fig. 1A and B). We next treated two additional murine p53^+/+^ cells lines with Navtemadlin to test whether the drug treatment was specific to B16-F10 cells. We observed significant cell growth arrest in the mouse melanoma cell line YUMM 1.7 (IC_50_ of 1.6 μmol/L; Fig. 1C) and in murine colon carcinoma cells, CT26.WT (IC_50_ of 2 μmol/L; Fig. 1D). Control murine fibroblast cells (NIH/3T3) were unaffected at the IC_50_ doses of the tumor cell lines, but displayed growth inhibition at high concentrations of Navtemadlin (4 and 8 μmol/L; Fig. 1E; Supplementary Fig. S2).

We next studied the cell cycle using EdU incorporation in both B16-F10 p53^+/+^ and p53^−/−^ cells. When treated with Navtemadlin at the IC_50_ concentration, p53^+/+^ cells exited S-phase and were arrested in the G_0_–G_1_ phase as early as 24 hours after treatment while p53^−/−^ cells were unaffected. We also detected an increase in the percentage of p53^+/+^ cells in sub-G_1_-phase at 48 and 72 hours (Fig. 1F–H). Thus, we found that Navtemadlin not only inhibits B16-F10 growth mainly through cell-cycle arrest, but also induces cell death in a population of cells in a p53-dependent manner.

### On-target Effects of Navtemadlin in B16-F10 Melanoma Cells

**FIGURE 2:**
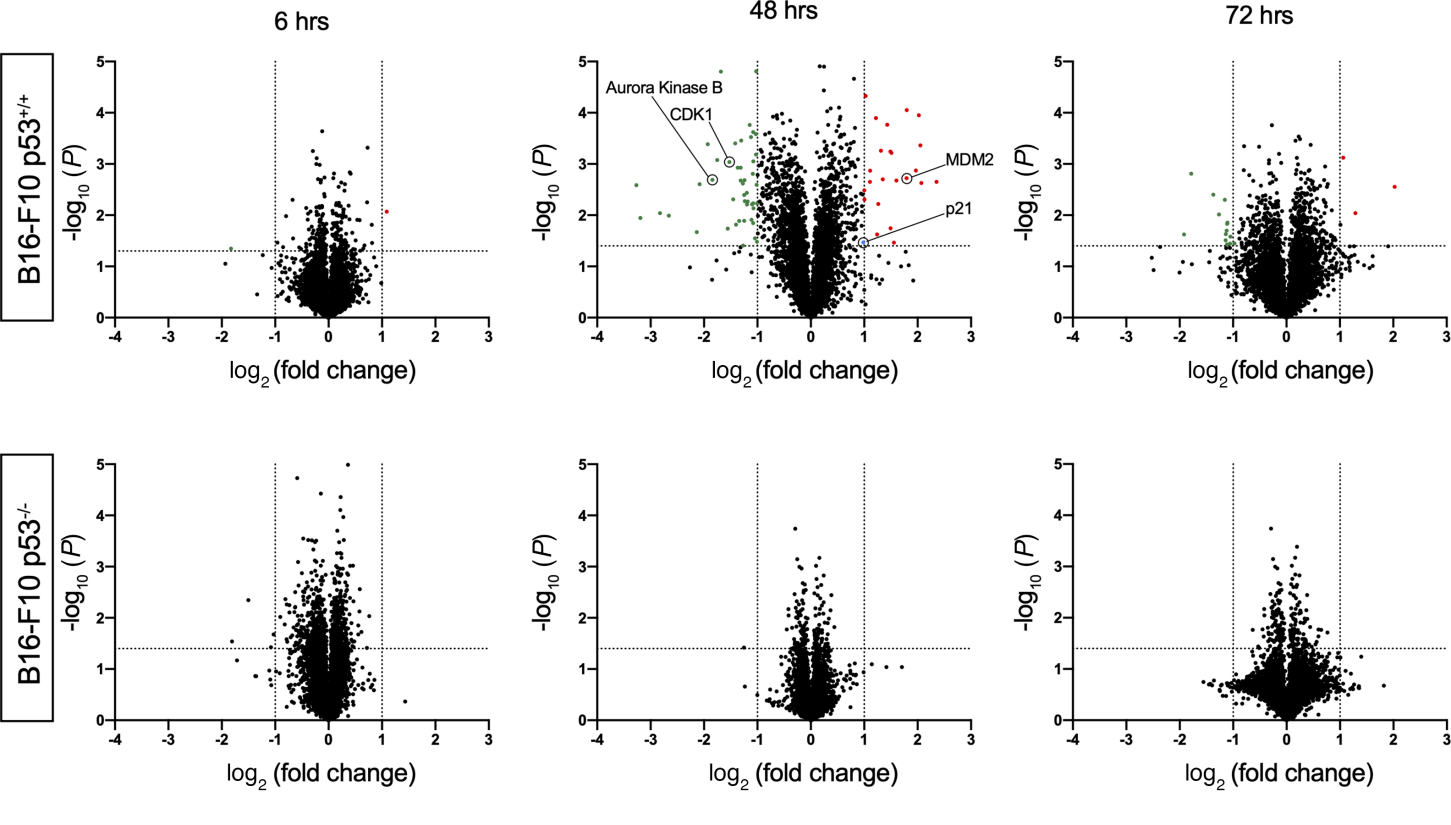
Proteomic analysis reveal on target nature of Navtemadlin. Changes in protein expression of B16-F10 p53^+/+^ (top) and p53^−/−^ (bottom) cells treated with 1.5 μmol/L Navtemadlin for 6, 48, and 72 hours was studied using mass spectrometry–based proteomics analysis. Proteins significantly upregulated are depicted in red and those that are downregulated in green. One experiment with triplicates.

Small-molecule drugs can have a wide range of off-target effects, which can affect the overall efficacy of the drug (29). Because Navtemadlin has been reported to be “best-in-class” p53-MDM2 PPI inhibitor, we used mass spectrometry-based proteomics to study changes in global protein expression in B16-F10 p53^+/+^ and p53^−/−^ melanoma cells treated *in vitro* with vehicle or Navtemadlin (1.5 μmol/L) for 6, 48, and 72 hours. We observed a significant upregulation and downregulation of proteins in p53^+/+^ cells, especially in the 48-hour treatment group (Fig. 2). These changes appear to be driven by reactivation of p53, for instance, upregulation of MDM2 and p21 and downregulation of AURORA KINASE B and CDK1. Remarkably, we did not observe any changes in protein expression in the p53^−/−^ cells treated with Navtemadlin at 48 and 72 hours. We however did observe four nonspecific proteins downregulated at the 6-hour timepoint in the p53^−/−^ cells, but these were not observed at the later timepoints tested (Supplementary Table S1 and see excel list in Supplementary Data for a complete list of proteins). These data indicate that Navtemadlin has very few or no off-target effects in contrast to the reported off-target effects of Nutlin-3 (8, 30).

### Navtemadlin Upregulates Key p53 Downstream Targets

**FIGURE 3:**
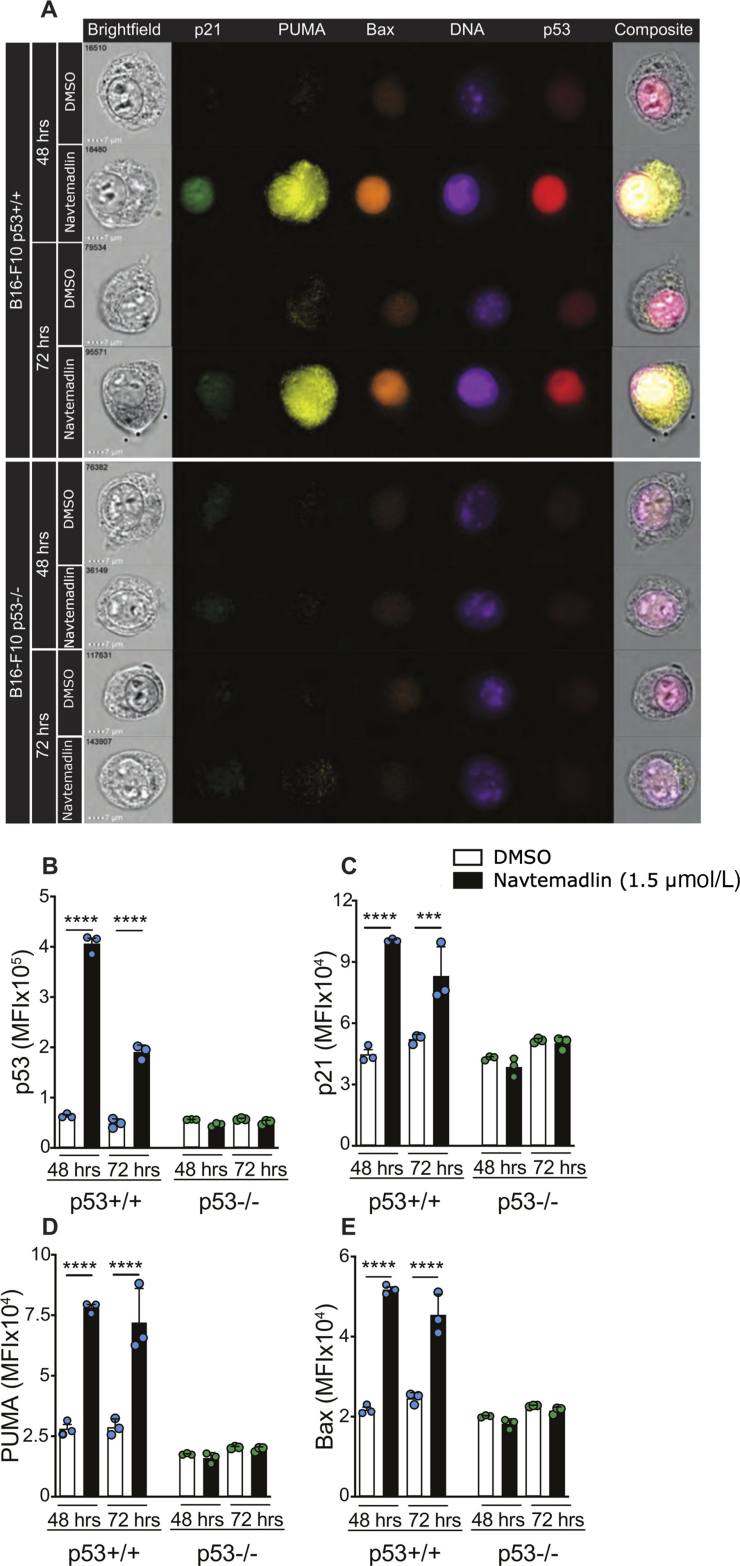
Imaging flow cytometry reveal Navtemadlin-dependent upregulation of downstream targets of p53. **A,** Representative imaging flow cytometry images of B16-F10 p53^+/+^ and p53^−/−^ cells treated with 1.5 μmol/L Navtemadlin or DMSO control for 48 and 72 hours. **B–E,** Median fluorescent intensity (MFI) of p53, p21, PUMA and BAX in B16-F10 cells. Data representative of two to three experiments performed with triplicates. Mean ± SD. ***, *P* < 0.0002; ****, *P* < 0.0001. One-way ANOVA.

Reactivation of p53 results in direct p53-related gene transcription of close to 118 genes (7). Because Navtemadlin induced cell-cycle arrest and specific protein upregulation in a p53-dependent manner in the B16-F10 cells, we next studied whether p53 downstream target proteins were upregulated. We used multi-color imaging flow cytometry, a method that combines fluorescent imaging and flow cytometry, to study the expression of p53 and selected direct downstream targets involved in cell-cycle arrest and apoptosis, that is, p21, BAX, and PUMA. We detected highest p53 expression in the nucleus of B16-F10 p53^+/+^ cells at 48 and 72 hours after Navtemadlin treatment. We also observed significantly higher expression of p21, BAX, and PUMA at 48- and 72-hour timepoints in Navtemadlin-treated B16-F10 p53^+/+^ cells compared with B16-F10 p53^−/−^ and control-treated cells (Fig. 3A–E). Furthermore, our data show that individual cells express p53, p21, PUMA, and BAX at the same time using high-throughput imaging flow cytometry analyses (Fig. 3A). B16-F10 p53^−/−^ cells, as expected, did not display any changes in the expression of these target proteins after Navtemadlin treatment. These data further indicate the on-target effects of Navtemadlin and validate imaging flow cytometry as a tool to study multiple protein expressions in a quantitative and high-throughput manner.

### Navtemadlin Potentiates Radiotherapy

Our data show that Navtemadlin induces p53 and its dependent downstream targets. However, we only observed a small portion of apoptotic cells following Navtemadlin treatment at IC_50_ concentration (1.5 μmol/L). This could be due to the fact that very high sustained levels of p53 are required to induce apoptosis, as shown in studies describing a threshold model for apoptosis (31). We therefore used live cell imaging to explore whether higher concentrations of Navtemadlin (3 and 6 μmol/L) increase levels of p53 and lead to more apoptosis in B16-F10 cells over time. Indeed, we observed a strong trend of higher p53 protein expression with Navtemadlin concentrations above the IC_50_ concentration (Supplementary Fig. S3A) and this increase in p53 correlated with an increase in apoptotic cells in culture. Interestingly, even at the highest concentrations tested (3 and 6 μmol/L), we only observed 35% apoptosis on average at 96 hours. (Supplementary Fig. S3B). To further understand the downstream proteins induced by Navtemadlin treatment, we used imaging flow cytometry to characterize the expression of p53 downstream targets: p21, BAX, and PUMA. We observed a significant increase in the expression of p21, BAX, and PUMA in Navtemadlin-treated p53^+/+^ B16-F10 cells compared with DMSO control, median fluorescence intensity measurement revealed that the expression of these downstream targets was not concentration dependent over the range of Navtemadlin doses tested (Supplementary Fig. S3C–E).

We hypothesized that the relative low percent of apoptotic cells in Navtemadlin-treated cells could be due to increased expression of survival signals in the B16-F10 p53^+/+^ cells. To test this hypothesis, we chose to study the expression of antiapoptotic proteins BCL-2 and MCL-1. We observed an increase in intracellular BCL-2 expression, however, to a lesser extent with the higher concentrations of Navtemadlin (Supplementary Fig. S3F). MCL-1 can bind to and inhibit proapoptotic protein PUMA (32, 33). However, it has been previously shown that phosphorylation of MCL-1 at Serine 159 (MCL-1-Ps159) leads to ubiquitination and degradation of MCL-1, promoting apoptosis (34, 35). Here, we used a mAb that specifically detects Serine 159 phosphorylation in MCL-1 protein. Navtemadlin-treated B16-F10 p53^+/+^ cells expressed significantly higher levels of MCL-1 with phosphorylated Serine 159 when compared with DMSO controls, but we did not observe a further increase in the amount of phosphorylated MCL-1 protein at higher concentrations of Navtemadlin (Supplementary Fig. S3G–H). These data suggest that Navtemadlin, when used as a monotherapy, can induce modest levels of apoptosis in B16-F10 melanoma cells because it induces both apoptotic and antiapoptotic proteins.

**FIGURE 4:**
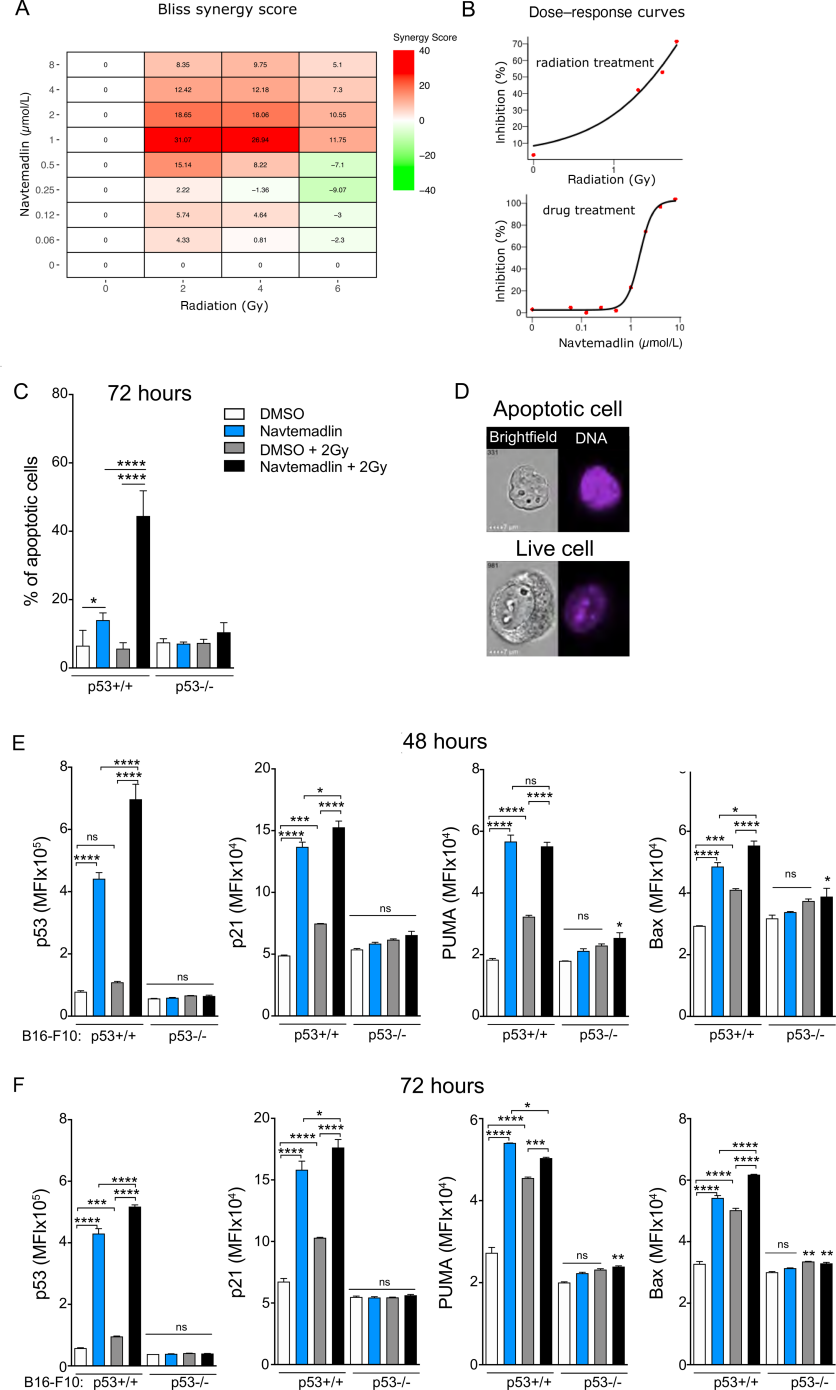
Reactivation of p53 with Navtemadlin synergizes with radiotherapy. **A,** B16-F10^+/+^ Bliss synergy scores for Navtemadlin and radiation combination treatments, BLISS score was defined as >10 synergistic (red), <−10 antagonistic (green), and <10 > −10 additive (white). **B,** B16-F10^+/+^ cell growth, measured as confluence by the Incucyte S3, % inhibited by radiotherapy and Navtemadlin treatment individually. **C,** % of apoptotic cells in B16-F10 cells treated with a single dose of 2 Gy radiation followed by 72 hours 1.5 μmol/L Navtemadlin. Apoptosis was defined by low nuclear area and high bright-field contrast using IDEAS software. **D,** Representative images of apoptotic and live B16-F10 p53^+/+^ cells were obtained after 72 hours combination treatment, 2 Gy followed by 1.5 μmol/L Navtemadlin treatment **E** and **F**, Median fluorescence intensity (MFI) of p53, p21, PUMA and BAX in B16-F10 cells analyzed with imaging flow cytometry after a single dose of 2 Gy radiation followed by 48 or 72 hours 1.5 μmol/L Navtemadlin treatment. Data in **C** pooled from two experiments performed with triplicates. Data in **A** and **B** and **E** and **F** representative of two to three experiments performed with triplicates. Mean ± SD. *, *P* < 0.05; **, *P* < 0.002; ***, *P* < 0.0002; ****, *P* < 0.0001. One-way ANOVA.

We next speculated that a combination therapy approach would be more effective in inducing higher rates of apoptosis. The use of external beam radiation in clinical cancer therapy is well established. Given the fact that p53 is activated because of cellular stress, including that induced by radiation, we next tested whether combining radiotherapy and Navtemadlin-mediated p53 reactivation would have a synergistic effect. B16-F10 p53^+/+^ and p53^−/−^ cells were first irradiated with a single dose of 2, 4, or 6 Gy radiation followed by Navtemadlin treatment. Cell growth was monitored using the IncuCyte S3 live imaging system. We observed a significant reduction in the growth of p53^+/+^ cells, but not in p53^−/−^ cells (Supplementary Fig. S4A). While the IC_50_ value of Navtemadlin alone was 1.5 μmol/L, combination with radiation resulted in lowering Navtemadlin IC_50_ by 2- to 5-fold (Supplementary Fig. S4B). However, p53^−/−^ cells appeared to be resistant to the combination of Navtemadlin and radiation treatment. To examine the interaction further, we calculated the Bliss synergy index landscape for the growth rates of the cells in the IncuCyte S3 system at each dose combination, using SynergyFinder. The results showed a clear synergistic peak in the growth rate reduction of Navtemadlin combined with radiation at 1 μmol/L and 2–4 Gy (Fig. 4A). The synergy index decreased above 1 μmol/L as well as above 4 Gy, this is however due to each individual treatment effect approaching 100% inhibition, as can be seen from the dose response curves (Fig. 4B), which prevents detection of synergy.

We next used imaging flow cytometry to study apoptosis induced by Navtemadlin as a monotherapy and in combination with radiation (2 Gy). We employed the IDEAS data analysis software where apoptotic cells are identified by lower nuclear area (nuclear stain FxCycle) and high bright-field contrast. Using this automated apoptosis analysis wizard, at the 72-hour timepoint, we observed a significant increase in the percentage of apoptotic cells when B16-F10 p53^+/+^ cells were treated with Navtemadlin + 2 Gy (Fig. 4C and D). Images taken during live cell imaging confirmed the appearance of enhanced cell death from the combination of Navtemadlin and 2 Gy radiation at the late timepoints 72 hours and increasing at 120 hours (Supplementary Fig. S5). To further understand the synergistic effects of Navtemadlin and 2 Gy radiation combination, we performed downstream target analysis using imaging flow cytometry. B16-F10 p53^+/+^ and p53^−/−^ cells were irradiated followed by Navtemadlin treatment (1.5 μmol/L). Cells were harvested after 48- and 72-hour treatment and stained for p53, p21, PUMA, BAX, and DNA intracellularly. The combination treatment mainly induced significantly higher p53 levels, but also modestly increased p21, and BAX levels in B16-F10 p53^+/+^, compared with single treatment or vehicle (Fig. 4E and F; Supplementary Fig. S6). Taken together, these data indicate that Navtemadlin potentiates radiotherapy in a p53-dependent manner.

### Navtemadlin Regulates Tumor Growth *In Vivo*

**FIGURE 5:**
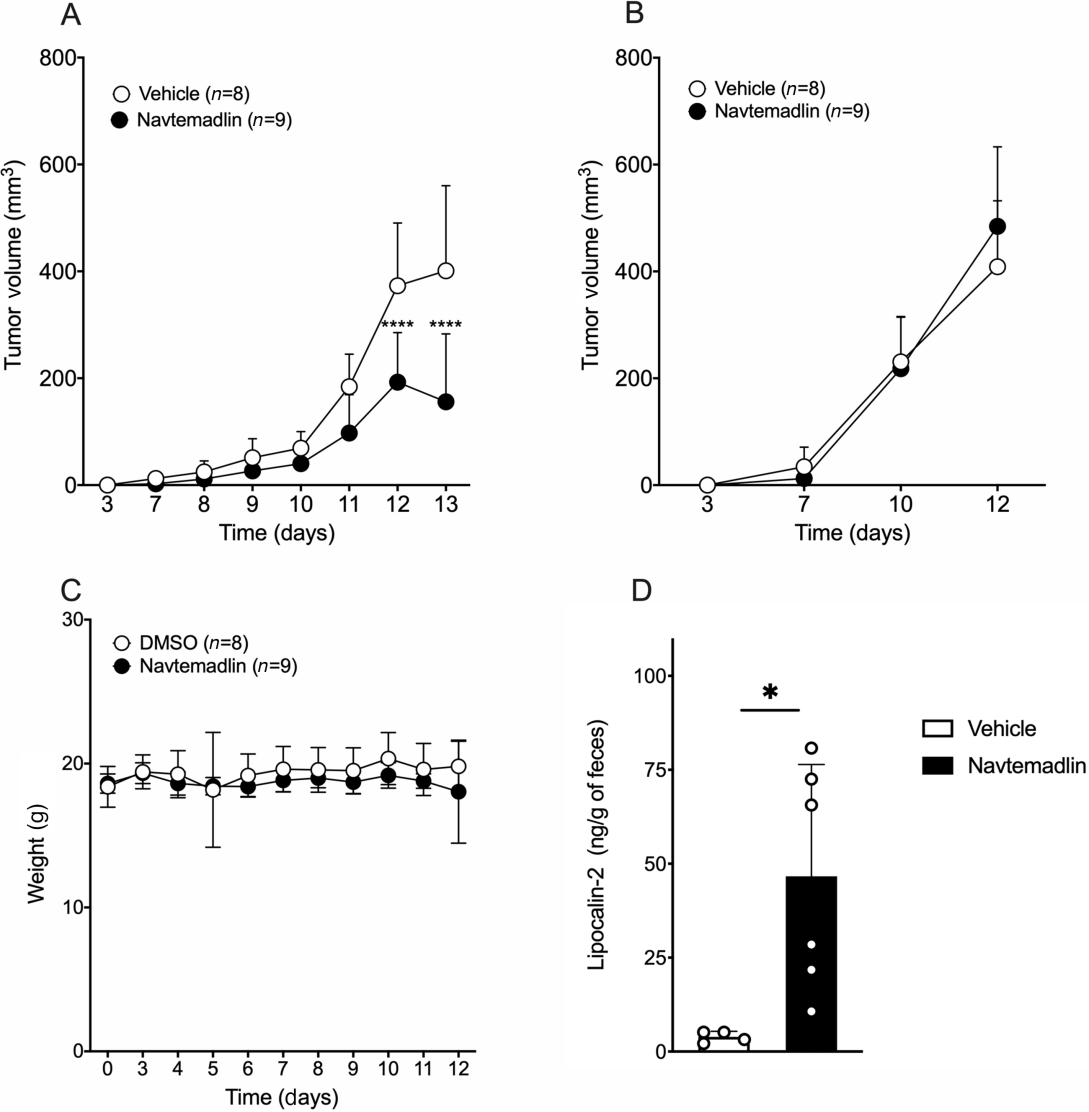
Navtemadlin induces tumor growth arrest *in vivo*. **A,** B16-F10 p53^+/+^ tumor volume (mm^3^). **B,** B16-F10 p53^−/−^ tumor volume (mm^3^). **C**, Mouse body weight (grams) at tumor implantation (day 0), before treatment start (days 0–3) and during treatment (days 3–12). **D,** LCN-2 concentration in feces after 10 days of treatment. Data representative of 1–2 experiments with 4–10 mice/group. Mean ± SD. ****, *P* < 0.0001. Two-way ANOVA.

To test whether our *in vitro* results using this small-molecule drug translate *in vivo*, we tested whether Navtemadlin treatment can impact tumor growth in a C57Bl/6 mouse implanted with B16-F10 melanoma tumors. A total of 10^5^ B16-F10 tumor cells in Matrigel were implanted subcutaneously to initate tumor development. A previously published study reported a dose-dependent effect on Navtemadlin on tumor reduction in human xenograft models using immunodeficient mice (13). Here we tested a low dose of Navtemadlin, 20 mg/kg (i.p.) and observed a reduction of around 30%–50% in p53^+/+^ tumor size when Navtemadlin was administered daily starting day 3 (Fig. 5A). However, Navtemadlin treatment did not affect the growth of B16-F10 p53^−/−^ tumors. (Fig. 5B). This lack of treatment effect in B16-F10 p53^−/−^ tumors led us to speculate that the main mechanism of p53^+/+^ tumor growth inhibition is a direct effect on the tumor cells themselves rather than an effect on the tumor microenvironment in this immunologically cold tumor model. We next confirmed this by investigating the tumor-infiltrating myeloid and lymphoid immune cell populations in the tumors after 10 days of treatment. We implanted p53^+/+^ and p53^−/−^ tumors subcutaneously in opposite flanks of the same mouse to test whether Navtemadlin treatment induces tumor immune cell infiltration. As we suspected, the infiltrating immune cell populations were not affected by treatment in either the B16-F10 p53^+/+^ or the p53^−/−^ tumors (Supplementary Fig. S7A and S7B, S8A and S8B). Importantly, this treatment regimen did not lead to bone marrow suppression (Supplementary Fig. S9A and S9B) or gastrointestinal symptoms such as diarrhea or weight loss in mice (Fig. 5C). However, fecal levels of the colitis marker LCN-2 were slightly enhanced in Navtemadlin-treated mice compared to control mice. This elevation of LCN-2 is not high enough to be consistent with clinical colitis though (36). These data show that Navtemadlin can induce tumor growth reduction *in vivo* in C57Bl/6 mice.

## Discussion

Reactivating WT p53 in cancers where the majority of the patients retain WT p53 is an attractive strategy to induce tumor cell death and regression (37). Melanoma is one such cancer where approximately 80% of the patients carry WT p53. Because cancers with WT p53 often have other mechanisms that suppress or inactivate the p53 response, primarily loss of p19^ARF^ and MDM2 amplification, inhibiting the p53-MDM2 interaction can be a beneficial strategy. In this study, we tested a “best-in-class” small-molecule MDM2 inhibitor, Navtemadlin which induces tumor growth reduction *in vitro* and in xenograft models in a dose-dependent manner (13). Several clinical trials testing the efficacy of Navtemadlin are currently underway. In a phase I study involving patients with solid tumors carrying WT p53, Navtemadlin demonstrated acceptable safety and dose-dependent pharmacokinetics (38). Despite the success of Navtemadlin in preclinical models and early clinical trials, it has been reported to be ineffective on mouse tumor cells, precluding its use in syngeneic mouse tumor models. However, such models are a valuable preclinical tool to simultaneously test the efficacy of a drug and its effects on tumors and the healthy tissue and in particular to examine the role of the immune system in tumor growth and response to therapy. Such models have been hard to establish with other MDM2 inhibitors due to their poor solubility and low-dose potency requiring the use of oral gavage. Indeed, most work has used the first-generation molecule Nutlin-3 which has limited dose potency and off target effects in p53 null cells including induction of the DNA damage response that greatly complicate interpretation of the *in vivo* results obtained with this agent (30).

Here, we tested whether reactivation of p53 using Navtemadlin in a mouse melanoma cell line, B16-F10, would induce tumor growth arrest and found that p53^+/+^ cells were indeed arrested in a dose-dependent manner. High concentrations of small-molecule drugs often tend to have off-target effects. Interestingly, the p53^−/−^ cells continued to grow, even at the highest concentration tested (8 μmol/L). When we repeated the experiment with two other p53 WT murine cell lines, YUMM 1.7 and CT26.WT, we obtained similar results. Navtemadlin, at 1 μmol/L, has been reported to arrest close to 80% of A375, human melanoma cells after 72 hours *in vitro* (13). Compared with these data, close to 75% of B16-F10 p53^+/+^ cells and YUMM 1.7 cells, were arrested at 2 μmol/L after 72-hour treatment. This comparison, although not performed in the same experiment, indicate that Navtemadlin is a potent MDM2 inhibitor in murine melanoma cells as well. The NIH/3T3 fibroblasts, which have WT p53, were also growth inhibited, but only at the highest concentrations of Navtemadlin tested (4 and 8 μmol/L), emphasizing that the response to Navtemadlin may vary with cell type as well as species.

Navtemadlin has been reported to have 40-fold less biochemical potency on murine MDM2; however, the murine cell lines tested or the experimental context have not been specified in this report (13). Because our *in vitro* experiments showed a potent growth inhibition and an IC_50_ of 1.5 μmol/L on the B16-F10 p53^+/+^ cells, close to the IC_50_ reported in human cells, we used mass spectrometry–based proteomics to identify global changes in the proteome of the B16-F10 cells following Navtemadlin treatment. Four proteins were detected as significantly downregulated at the 6-hour timepoint in the B16-F10 p53^−/−^ cells. The identified proteins are not known to have any role in the p53 pathway. However, MDM2 has been reported to bind directly to chromatin and to interfere with amino acid metabolism (39). It is therefore possible that these four proteins could be downregulated due to MDM2 inhibition in the p53^−/−^ cells. Although these proteins are detected at 6 hours, the effect appears to be transient, as no other proteins were significantly downregulated or upregulated in the knockout cells at the other timepoints tested, indicating that Navtemadlin has almost no off-target effects in mouse cells, and the effects observed in the p53^+/+^ cells are dependent on WT p53 expression. Among the p53^+/+^ cells, proteins that are downstream targets of p53 (such as MDM2 and p21) were upregulated, while proteins essential for the progression of the cell cycle were downregulated. Mass spectrometry–based proteomics can therefore be very valuable in understanding the effects of cancer drugs, particularly those that target crucial pathways such as p53. However, this approach may not be effective in identifying subtle changes in protein expression as they may be drowned by the signal from proteins that are highly expressed. We therefore employed imaging flow cytometry, a method that combines high-throughput flow cytometry with microscopy enabling quantitative studies. We developed a panel by combining five fluorescent markers together with bright-field images to assess key downstream targets of p53 involved in cell-cycle arrest and apoptosis. We were not only able to detect the expression of p21, BAX, and PUMA in p53^+/+^ cells that expressed high levels of p53, but also detect their subcellular localization in a large number of cells sampled per replicate. Importantly, our analysis also revealed the simultaneous expression of p21, BAX, and PUMA at a single-cell level in which we were also able to evaluate p53 expression and apoptosis. To our knowledge, the single-cell coexpression of CDK inhibitor p21 and proapoptotic proteins such as PUMA and BAX has not been reported before.

Although Navtemadlin induced potent cell-cycle arrest in B16-F10 p53^+/+^ cells, we only detected a small portion of these cells undergoing apoptosis, even after 72-hour treatment. The population of apoptotic cells increased with higher concentration of Navtemadlin and stronger p53 induction, especially at the 96-hour timepoint. The expression of p21 or proapoptotic proteins PUMA and BAX did however not change with Navtemadlin concentration. The results support the findings of Kracikova and colleagues (31) that the induction of apoptosis as opposed to growth arrest by p53 requires a higher and more sustained level of p53 activity and is not caused by differences in the threshold for induction of p21 and PUMA. Induction of a stronger p53 response may flip cells across the apoptotic threshold. To achieve an apoptotic response instead of a reversible cell-cycle arrest, it may therefore be crucial to reach this threshold of p53 protein concentration. We also discovered that the expression of the antiapoptotic protein BCL-2 increased when we treated the B16-F10 p53^+/+^ cells. We detected a strong signal from phosphorylated MCL-1 after treatment. As these proteins are strong survival signals their expression may raise the apoptotic threshold, explaining why the majority of cells are G_1_ arrested with treatment. Radiotherapy is used in combination with other treatment strategies to manage melanoma in many patients (40). As radiation is also a known inducer of DNA damage and p53 responses, we chose to combine radiotherapy with Navtemadlin. Furthermore, Navtemadlin has been reported to have synergistic effects when combined with radiotherapy in a patient-derived xenograft model of adenoid cystic carcinoma (41). Our *in vitro* experiments show that Navtemadlin synergizes with radiation leading to increased apoptosis in B16-F10 p53^+/+^ cells compared with Navtemadlin or radiation as single agents.

The remarkable selectivity of Navtemadlin seen in our proteomic analysis is very important. A review of recent clinical trials using other small-molecule inhibitors of MDM2 suggests that a late hematological toxicity is an “on target” and inescapable side effect of MDM2 inhibitory drugs (42). In sharp contrast, the trial of a stapled peptide inhibitor showed clear efficacy but no such hematologic effect (43) , suggesting that off-target effects or differential drug distribution may be the cause of hematologic toxicity. If so, the new generation of much more potent and selective inhibitors represented here by Navtemadlin may have superior efficacy. Our results suggest however that the dosing regime needs to recognize the need for a sustained p53 response to cross the apoptotic threshold and that local radiation may assist with this while minimizing systemic side effects.

Human xenograft models treated with Navtemadlin were shown to have a dose-dependent response to drug treatment (13). We used a syngeneic mouse tumor model to assess whether Navtemadlin can inhibit mouse tumor growth *in vivo*. When administered intrapeitoneally, daily injections of Navtemadlin induced a significant p53-dependent growth arrest at a 20 mg/kg dose. Interestingly, xenograft models using human melanoma cells had a ED_50_ of 18 mg/kg when administered orally (13). We found that the inhibition of tumor growth *in vivo* was a direct result of the growth arrest on the B16-F10 tumor cells and was not enhanced by effects on the tumor microenvironment in this model. The main side effect reported from clinical trials evaluating Navtemadlin, has been gastrointestinal symptoms and Neutropenia (44, 45). Hence, we investigated the potential side effects of Navtemadlin on mice. Treatment with 20 mg/kg did not result in bone marrow suppression or gastrointestinal symptoms such as diarrhea. It also did not result in changes in mouse weight. However, when we measured the fecal concentration of the colitis marker LCN-2, we found slightly increased concentrations in the treated mice compared with DMSO-treated control mice. A previous study have shown that fecal concentration of LCN-2 needs to be elevated around 10,000 fold to result in clinical colitis detected by histopathologic analysis (36). In our study, we detected far lower concentrations which may be the sign of low-grade inflammation or a prestage of colitis that could develop with time. However, we conclude that after 10 days of treatment the mice did not show any clinical signs of gastrointestinal symptoms. Our results therefore indicate that Navtemadlin is also an efficient inhibitor of murine MDM2, in contrast to what has been previously reported.

In summary, here we report for the first time that the p53-MDM2 inhibitor Navtemadlin efficiently inhibits mouse melanoma tumor cell growth *in vitro* and *in vivo* in a p53-dependent manner. We also demonstrate the use of proteomics and imaging flow cytometry for evaluating drug-induced changes in protein expression. It is known that the B16-F10 melanoma tumors grow rapidly after around 9 days of tumor implantation and at this point, the tumors do not respond to monotherapy, including checkpoint immunotherapy. Our syngeneic B16-F10 p53^+/+^ mouse melanoma model can be used to test existing p53-MDM2/MDM4 inhibitors and to understand how they regulate tumor growth. Furthermore, this model can be used to identify new combination therapies that can efficiently eliminate tumors *in vivo*. The model will also allow examination of the threshold model for apotosis induction by p53 activation as we can directly study p53 levels in the tumor before and after treatment.

## Supplementary Material

Supplementary Data Complete list of proteins.

Figure S1 Crispr deletion of p53 in clone 8.

Figure S2 Incucyte images.

Figure S3 B16-F10 +/+ cells treated with increasing Navtemadlin concentrations over time.

Figure S4 Navtemadlin treatment potentiates radiotherapy in p53+/+ B16-F10 melanoma cells.

Figure S5 Images radiation + Navtemadlin combination.

Figure S6 Flow data radiation + Navtemadlin combination.

Figure S7 Navtemadlin treatment does not affect tumor infiltrating myeloid cells.

Figure S8 Navtemadlin treatment does not affect tumor infiltrating lymphocytes.

Figure S9 Navtemadlin treatment does not lead to bone marrow suppression.

Table S1 Proteomics data.
