# Supplementary figures and images for: The MDM2 Inhibitor Navtemadlin Arrests Mouse Melanoma Growth In Vivo and Potentiates Radiotherapy
Source: Cancer Res Commun. 2022 Sep 28;2(9):1075–88. doi: 10.1158/2767-9764.CRC-22-0053 (PMC10010373; doi:10.1158/2767-9764.CRC-22-0053)

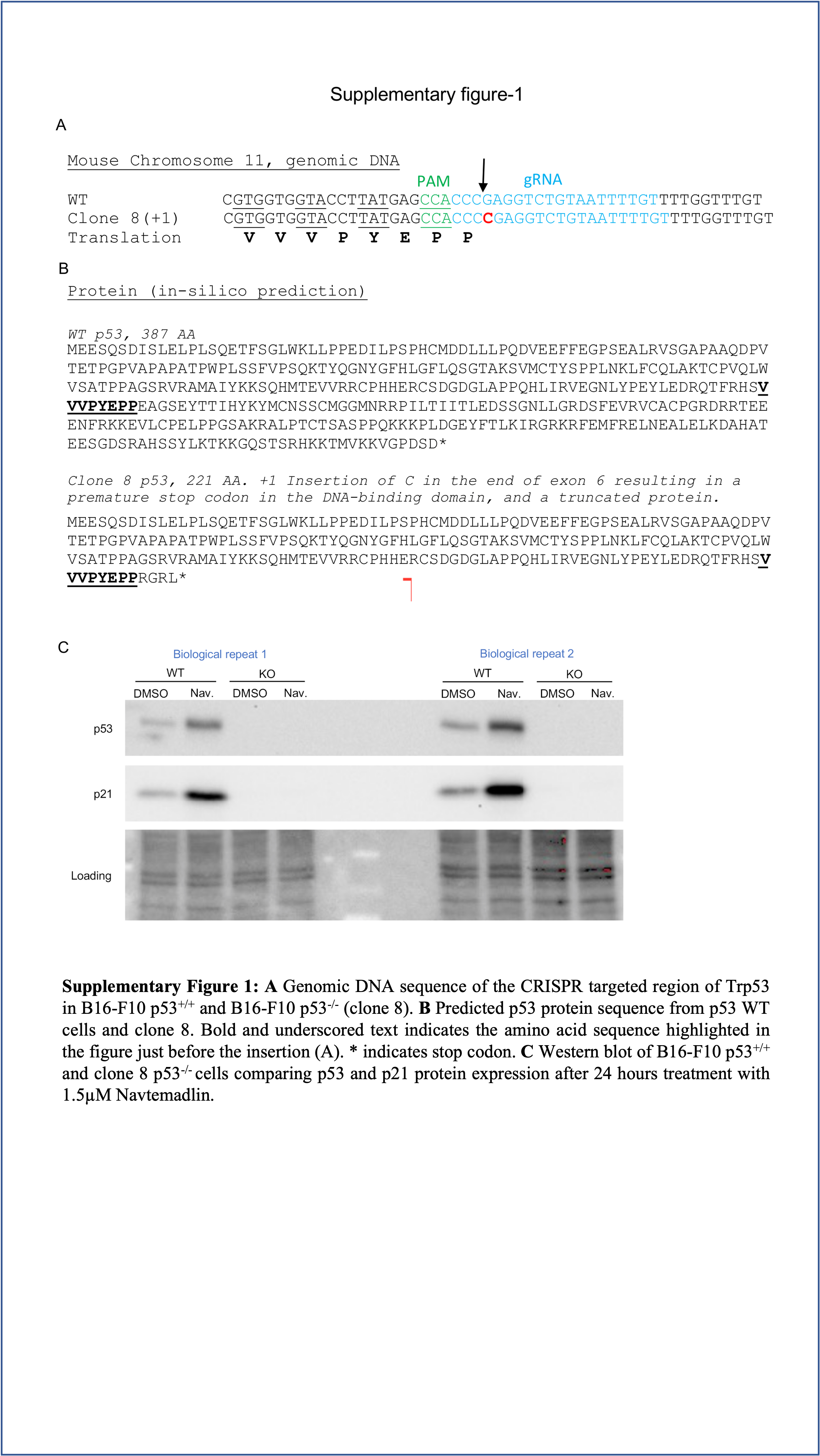

Supplement: Figure S1 — Crispr deletion of p53 in clone 8. [file crc-22-0053-s02.png]

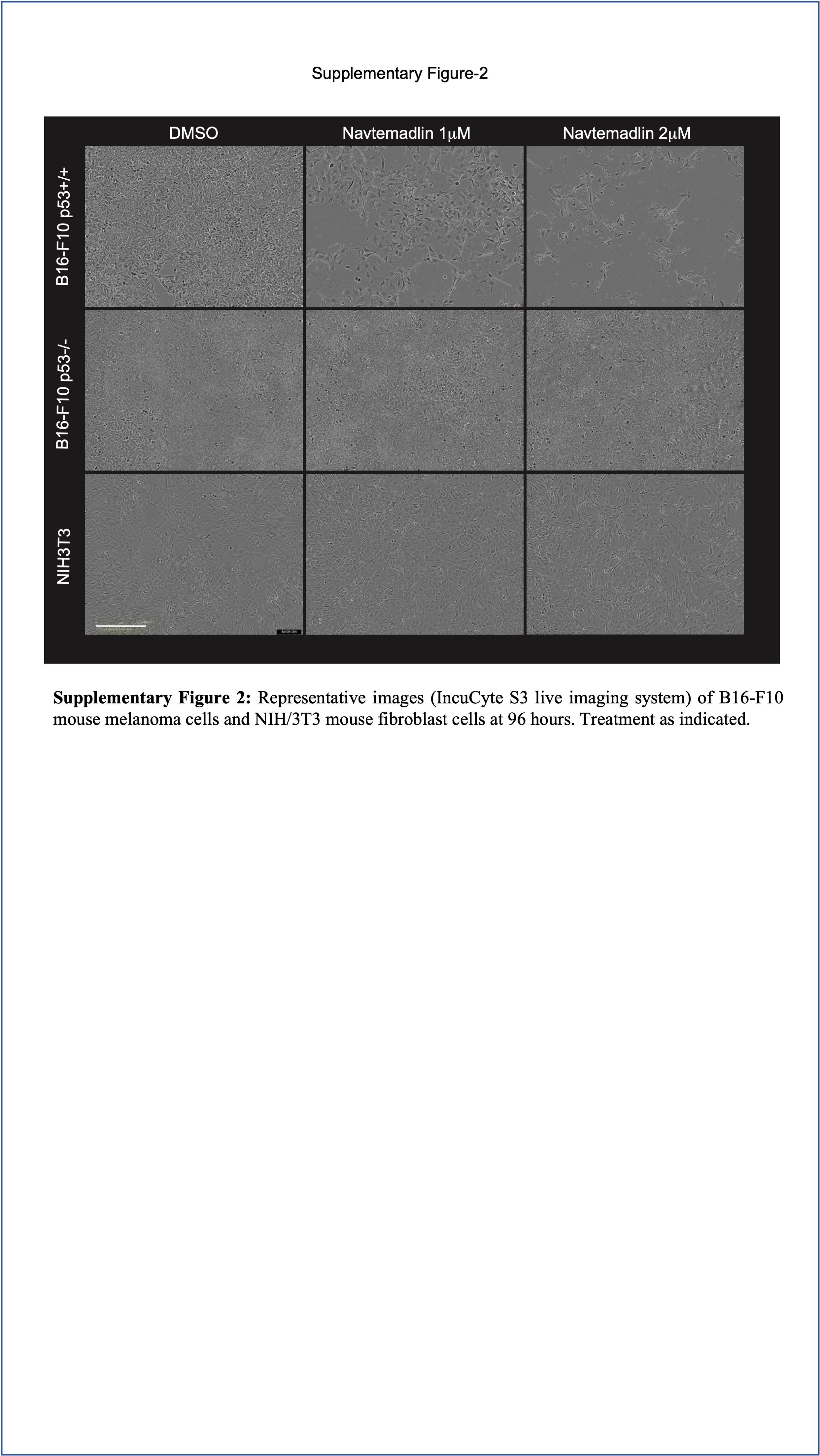

Supplement: Figure S2 — Incucyte images. [file crc-22-0053-s03.png]

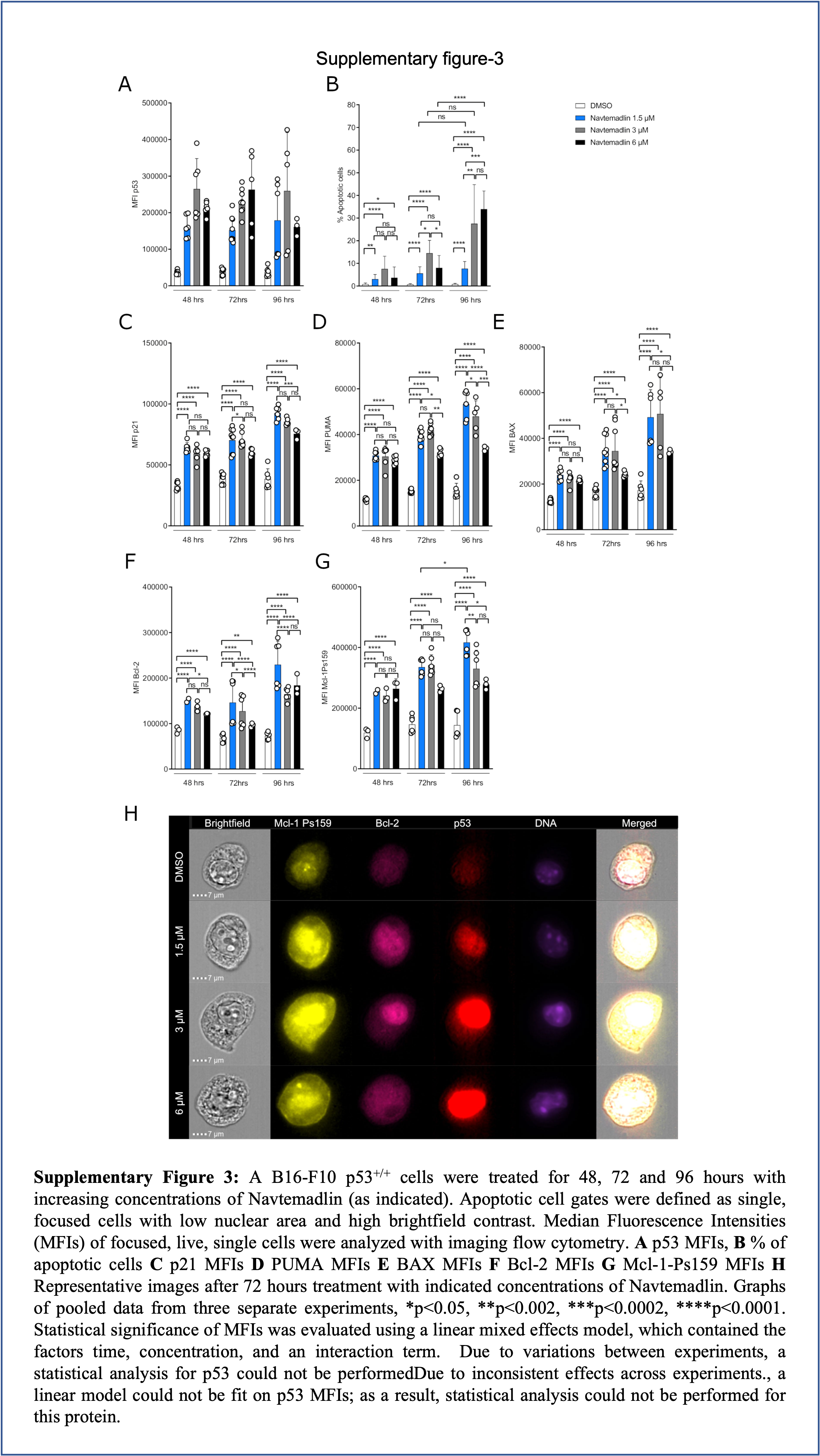

Supplement: Figure S3 — B16-F10 +/+ cells treated with increasing Navtemadlin concentrations over time. [file crc-22-0053-s04.png]

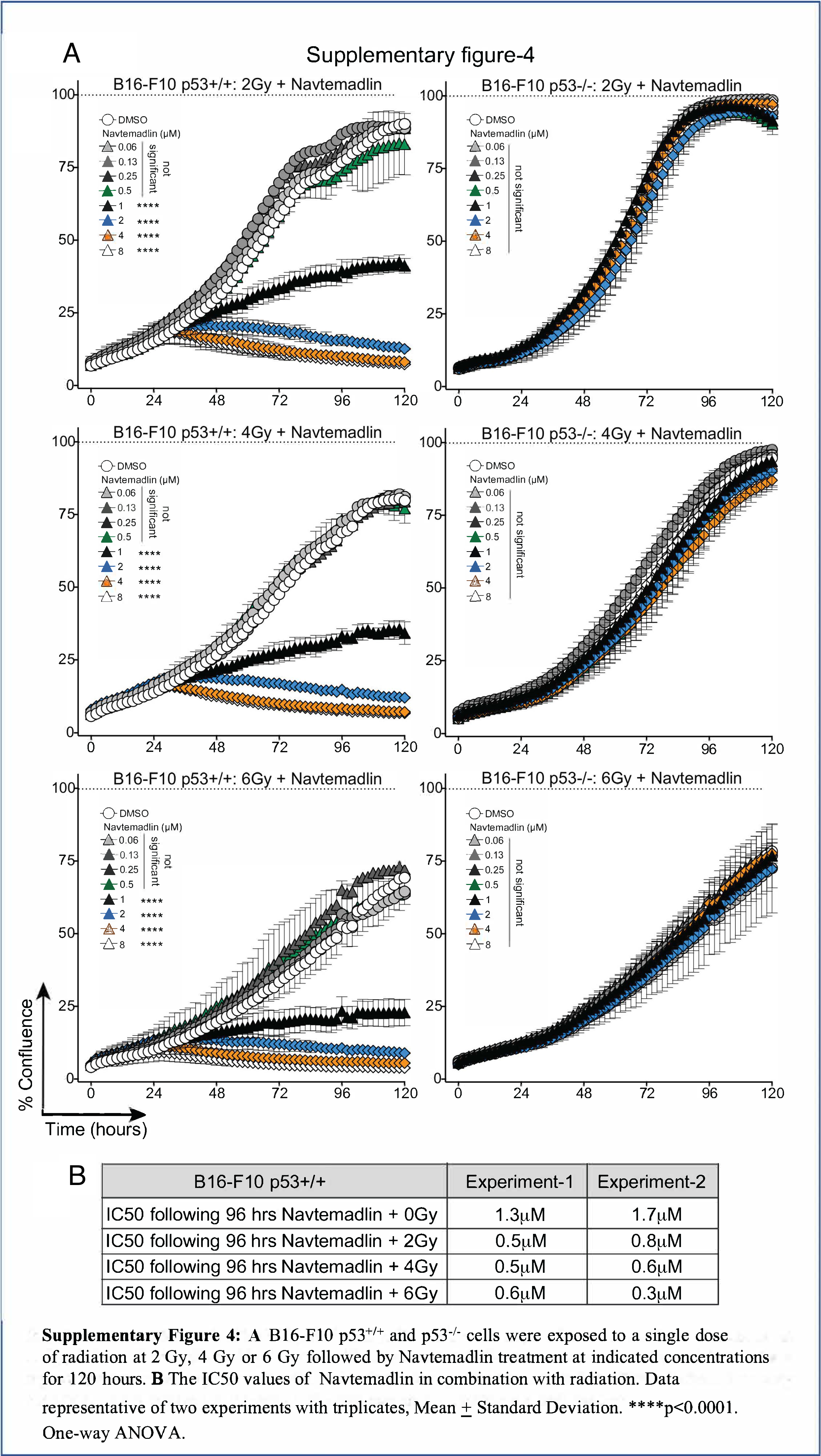

Supplement: Figure S4 — Navtemadlin treatment potentiates radiotherapy in p53+/+ B16-F10 melanoma cells. [file crc-22-0053-s05.png]

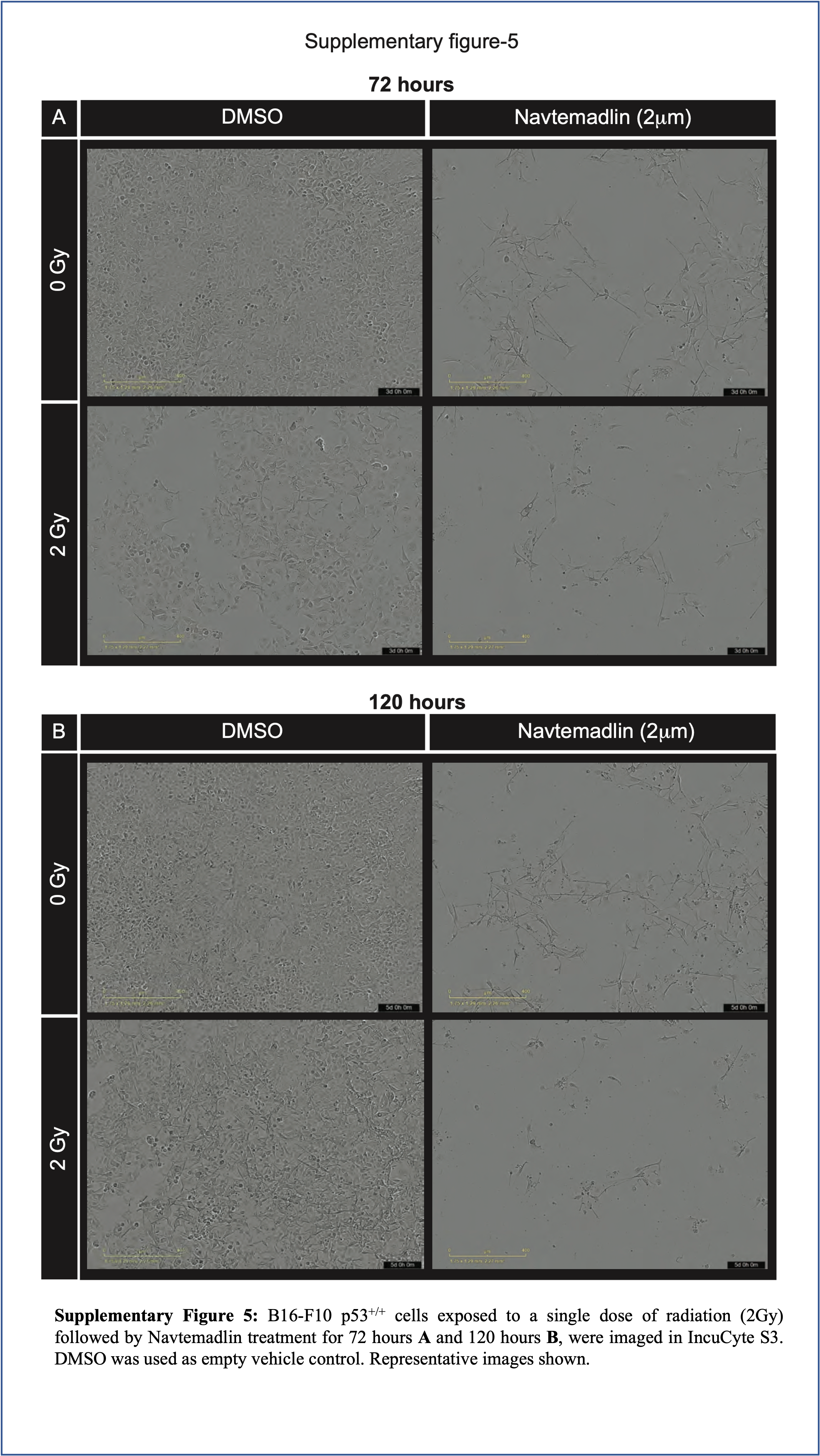

Supplement: Figure S5 — Images radiation + Navtemadlin combination. [file crc-22-0053-s06.png]

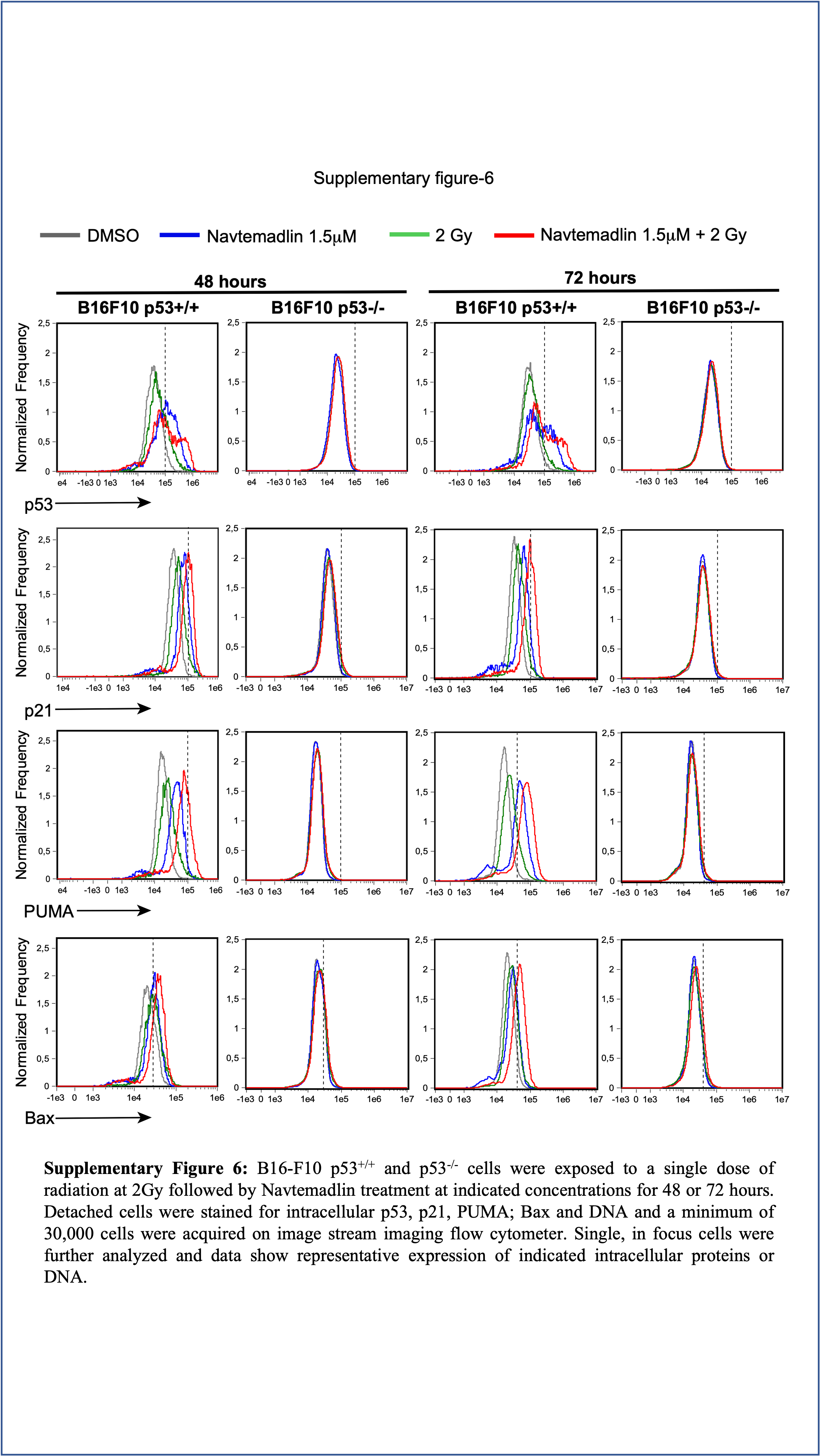

Supplement: Figure S6 — Flow data radiation + Navtemadlin combination. [file crc-22-0053-s07.png]

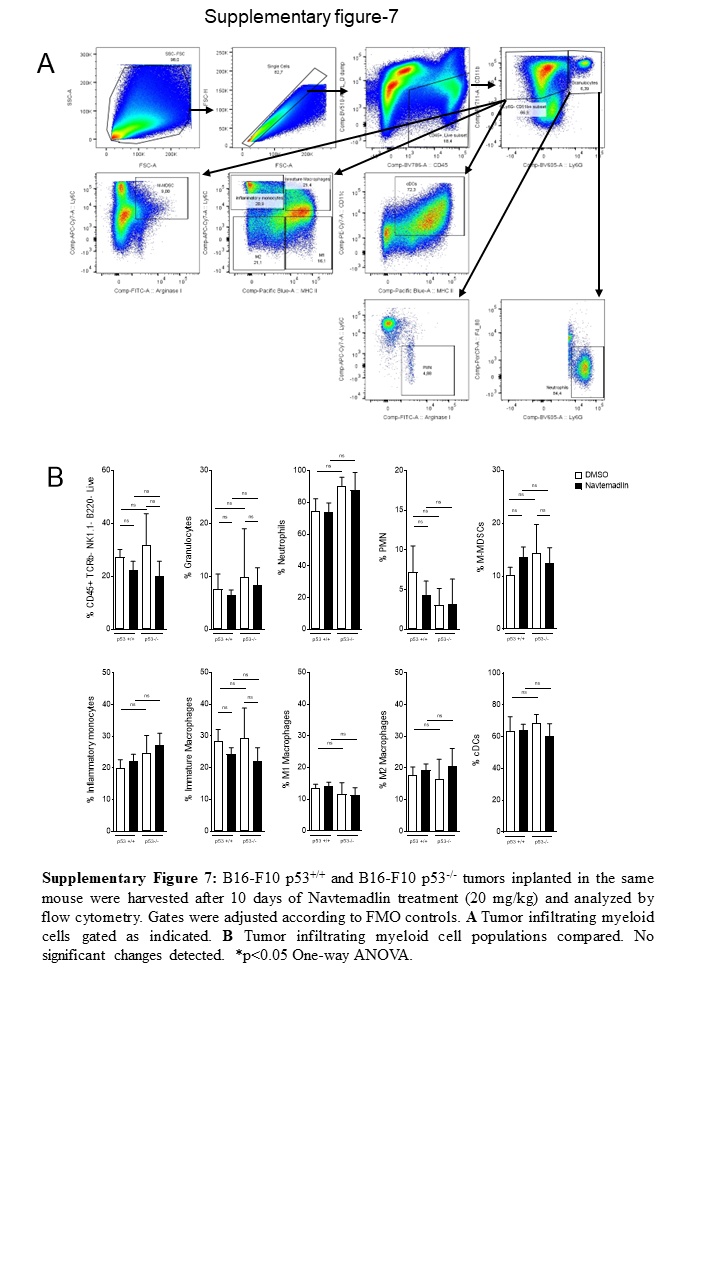

Supplement: Figure S7 — Navtemadlin treatment does not affect tumor infiltrating myeloid cells. [file crc-22-0053-s08.png]

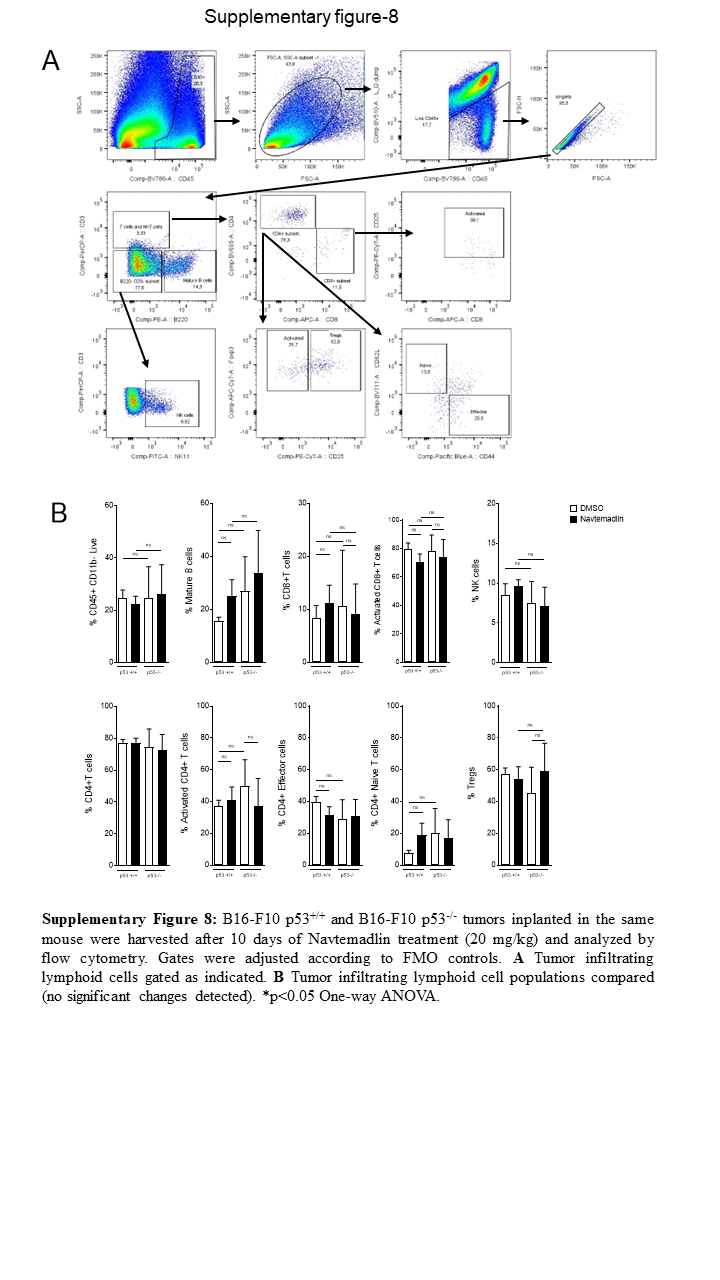

Supplement: Figure S8 — Navtemadlin treatment does not affect tumor infiltrating lymphocytes. [file crc-22-0053-s09.png]

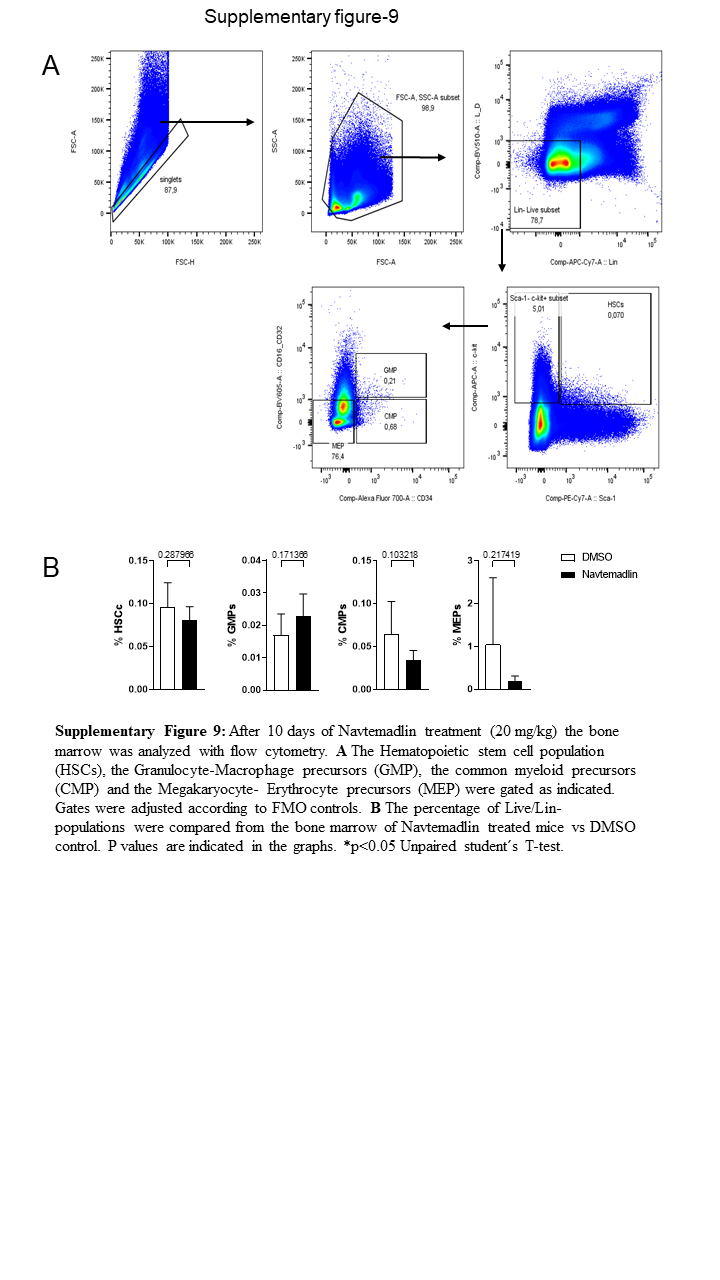

Supplement: Figure S9 — Navtemadlin treatment does not lead to bone marrow suppression. [file crc-22-0053-s10.png]

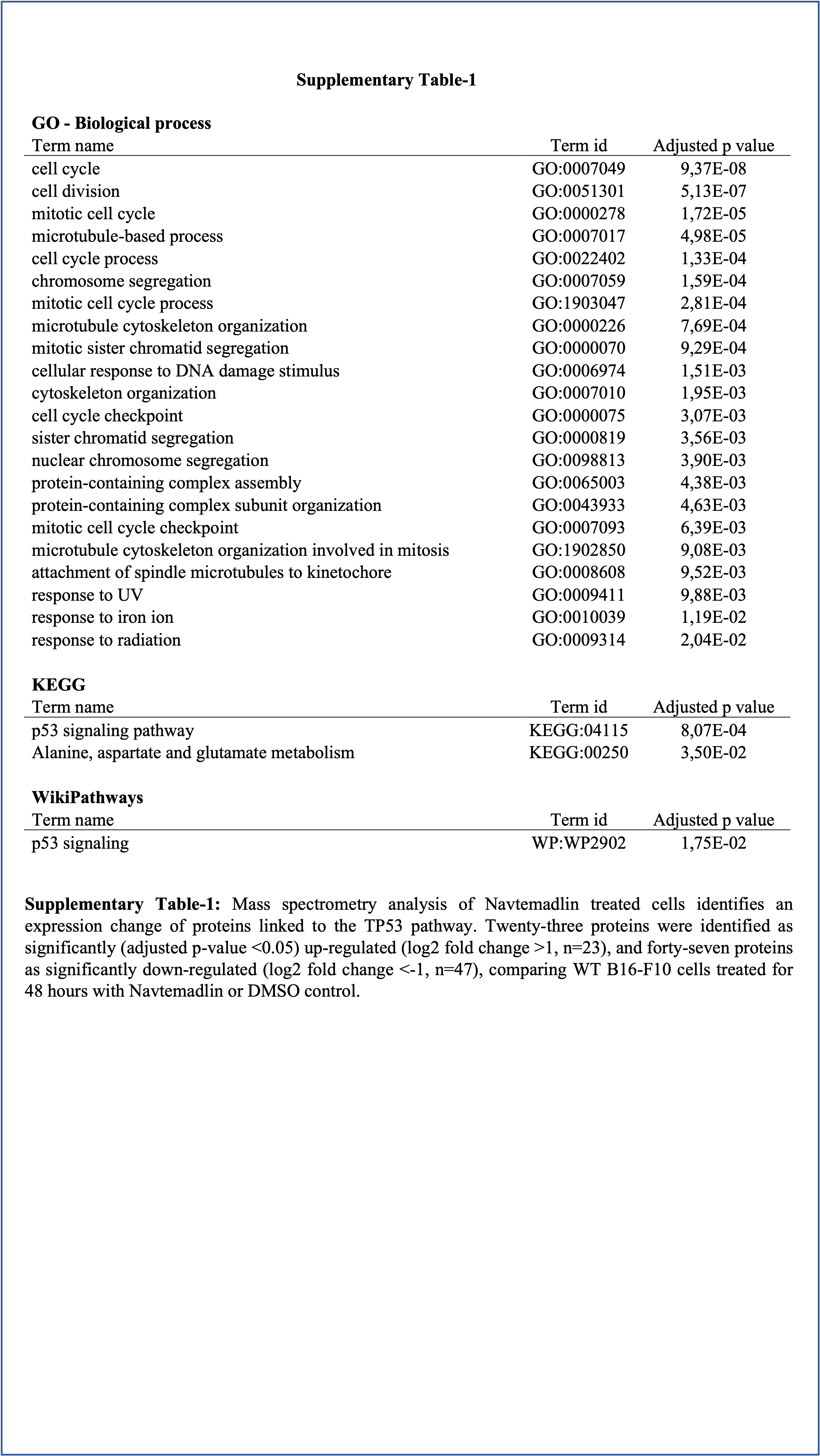

Supplement: Table S1 — Proteomics data. [file crc-22-0053-s11.png]
